# Acute fatigue in indoor court-based team sports: A systematic review

**DOI:** 10.1371/journal.pone.0316831

**Published:** 2025-02-14

**Authors:** Anthony Clark, Omar Heyward, Lara Paul, Ben Jones, Sarah Whitehead

**Affiliations:** 1 Carnegie School of Sport, Leeds Beckett University, Leeds, United Kingdom; 2 Leeds Rhinos Netball, Leeds, United Kingdom; 3 Rugby Football Union, London, United Kingdom; 4 Division of Physiological Sciences and Health through Physical Activity, Lifestyle and Sport Research Centre, Department of Human Biology, Faculty of Health Sciences, University of Cape Town, Cape Town, South Africa; 5 England Performance Unit, Rugby Football League, Leeds, United Kingdom; 6 School of Behavioural and Health Sciences, Faculty of Health Sciences, Australian Catholic University, Brisbane, Queensland, Australia; 7 Premiership Rugby, London, United Kingdom; University of Vic - Central University of Catalonia: Universitat de Vic - Universitat Central de Catalunya, SPAIN

## Abstract

Fatigue in team sports has been widely researched, with a number of systematic reviews summarising the acute (i.e., within 48-hours) response in outdoor sports. However, the fatigue response to indoor court-based sports is likely to differ to outdoor sports due to smaller playing fields, harder surfaces, and greater match frequencies, thus should be considered separately to outdoor sports. Therefore, this study aimed to conduct a systematic review on acute fatigue in indoor court-based team-sport, identify methods and markers used to measure acute fatigue, and describe acute fatigue responses. A systematic search of the electronic databases (PubMed, SPORTDiscus, MEDLINE and CINHAL) was conducted from earliest record to June 2023. Included studies investigated either a physical, technical, perceptual, or physiological response taken before and after training, match, or tournament play. One-hundred and eight studies were included, measuring 142 markers of fatigue. Large variability in methods, fatigue markers and timeline of measurements were present. Cortisol (n =  43), creatine kinase (n =  28), countermovement jump (n =  26) and testosterone (n =  23) were the most frequently examined fatigue markers. Creatine kinase displayed the most consistent trend, increasing 10–204% at 24-hours across sports. There is large variability across studies in the methods and markers used to determine acute fatigue responses in indoor court-based team sports. Future researchers should focus on markers that display high reliability and transfer to practice. The robustness of studies may be increased by ensuring appropriate methods and timescale of fatigue marker measurement are used. Further research is required to determine which combination of markers best describes a fatigue response.

## 1. Introduction

Acute fatigue is a critical factor influencing the performance and well-being of athletes in various sports disciplines, due to its impact on physical performance, athlete motivation, and injury risk [1–3]. Indoor court-based sports (e.g., basketball, volleyball, netball) are characterised by smaller playing fields, harder surfaces, movement constraints, and shorter match durations than outdoor sports [4–6]. These factors impose specific demands on athletes, requiring quick decision-making and frequent high-intensity efforts including frequent accelerations, decelerations, jumps, and changes-of-direction [7–11]. These dynamic movements have a high eccentric load, which combined with hard playing surfaces, may result in greater exercise induced muscle damage and fatigue accumulation than similar actions performed on softer surfaces [12–14]. Furthermore, sustained high-intensity efforts lead to depletion of energy stores and an increase in metabolic by-products such as reactive oxygen species (ROS) and inorganic phosphate which may disrupt cellular function and impact subsequent performance [15–18]. Understanding the impact of these factors on fatigue within indoor team sport athletes is crucial for developing targeted training and recovery strategies to optimise performance, reduce injury risk, and enhance overall well-being [19,20].

Acute fatigue may be defined as a psychophysiological condition characterised by a decline in motor or cognitive performance and/or an increased perception of tiredness or exhaustion [18,21]. Acute fatigue typically persists for a duration ranging from a few hours to several days and is recognised as a necessary component of the training-adaptation process [22,23]. However, mismanagement of acute fatigue can have detrimental effects, leading to compromised competitive performance and increased risk of injuries [1,3,18]. Furthermore, the cumulation of fatigue over an extended period (i.e., weeks to months), poses the risk of non-functional overreaching which may in turn lead to conditions such as overtraining syndrome [24]. These factors may be further exacerbated in indoor court-based team sports which commonly include congested competition schedules (e.g., matches every two to three days), that provide inadequate time to recover between matches, and long seasons lasting six months or more, that increase the risk of fatigue cumulation [25]. Given the multi-factorial nature of acute fatigue, measurements across the domains of physical performance, physiology, cognitive function, and perceived fatigue/wellness are required to comprehensively understand it.

Consequently, numerous investigations have been conducted to explore the acute fatigue response in team sports, encompassing both indoor and outdoor disciplines [26–31]. These studies have predominantly focused on assessing fatigue in the context of match play [32,33], training [29,34], and field or laboratory-based sport simulation protocols [28,35,36]. However, it is important to acknowledge the inherent limitations associated with conducting research in team sporting environments such as staff buy in and funding [37], and constraints in the number and types of fatigue measurements employed in each sport. Moreover, while sport simulation protocols provide controlled conditions, they fail to replicate the technical and tactical stressors inherent in actual sporting play, potentially influencing the observed fatigue response [38].

In a previous review by Doeven et al. [39], the post-match recovery timeline of team ball-sport athletes was investigated, revealing substantial variability in fatigue markers across different sports, both within and between markers. The countermovement jump (CMJ) emerged as the most frequently evaluated physical performance marker, while creatine kinase concentration ([CK]) was the predominant biochemical marker, with both markers typically peaking within 24-hours post-match [39]. Furthermore, physical markers of fatigue such as the CMJ tended to resolve within 48-hours while biochemical markers such as [CK] were more varied and remained elevated for over 72-hours in several studies [39]. However, this review encompassed a wide range of indoor and outdoor sports, potentially influencing the observed variation and did not include any measures of perceptual fatigue.

To date, the only review focusing specifically on indoor sports examined elite athletes over a period of four weeks or longer (i.e., longitudinal fatigue) and did not encompass popular indoor team sports such as handball and netball (25). No existing reviews have comprehensively examined the acute fatigue responses of indoor court-based team sport athletes across the domains of physical, physiological and perceptual fatigue. A review of this nature will enable sports practitioners to better understand the responses of athletes to matches and training, prescribe appropriate training loads and implement recovery strategies. Therefore, this systemic review aims to summarise the literature on acute fatigue in indoor court-based team sports, identify the methods and markers used to measure acute fatigue in these sports, and describe the acute fatigue in response to sport participation (i.e., match play, sports-specific training and tournaments) experienced by athletes in each sport.

## 2. Methods

### 2.1. Design and search strategy

This systematic review was conducted in accordance with the Preferred Reporting Items for Systematic reviews and Meta-Analyses (PRISMA) guidelines [40]. Prior to the commencement of the study, the review protocol was registered on the Open Science Framework (https://doi.org/10.17605/OSF.IO/EF3A9)*.*

The Population-Intervention-Comparators-Outcomes model (PICO) was used to guide the study eligibility criteria and assist in developing the search terms; *population*: indoor court-based team sport athletes, *intervention*: match play, tournament, or training participation, *comparators:* changes from pre-participation baseline values to measures taken within 48-hours of participation, *outcomes:* physical and technical performance, perceptual responses, biochemical and physiological parameters. The timeframe of 48-hours post sport participation was used as disturbances to fatigue measures have previously been shown to peak within 24 to 48-hours of match participation [39].

A systematic search of the literature was performed with the electronic databases PubMed and EBSCO host (SPORTDiscus, MEDLINE and CINHAL) was performed from the earliest record to 12 June 2023. The titles, abstracts, and keywords of articles in each database were searched using terms related to fatigue and court-based team sports in accordance with previous reviews on fatigue in sport [39,41,42]. The search terms are presented in Table 1, the terms from ‘Search 1’ were combined with ‘Search 2’ using the operator term AND to give the final search. Mental fatigue, alternatively termed cognitive or psychological fatigue, was not included within the search terms as its induction seems largely dependent on cognitive, rather than physical, tasks [18] and it has previously been examined in several reviews [38,43–45]. Reference lists of selected papers were manually searched for other potential eligible papers. The search results were then inserted in the Rayyan web application (https://rayyan.qcri.org).

**Table 1:** Search terms used to identify studies via the databases.

Search 1	Search 2
((fatigue) OR (recovery) OR (perceptual) OR (neuromuscular) OR (muscular power) OR (jump) OR (sprint) OR (agility) OR (technical) OR (metabolic) OR (physiological) OR (hormones) OR (muscle damage) OR (oxidative stress) OR (inflammation) OR (immunology))	((court sport) OR (indoor sport) OR (netball) OR (basketball) OR (floorball) OR (futsal) OR (handball) OR (indoor hockey) OR (korfball) OR (volleyball) OR (tchoukball))

### 2.2. Study selection

After eliminating duplicates, search results were independently screened by two researchers (AC and LP) against the eligibility criteria. Articles which could not be eliminated by title or abstract were retrieved and evaluated for inclusion via full text review. Disagreements between the two authors were resolved through discussion via a third researcher (SW).

To be included in the review studies had to: 1) be an original peer reviewed research paper that investigated an acute fatigue response, within 48-hours of participation, in an indoor team sport, played on a hard-wood or hard-court surface and 2) include either a physical, technical, perceptual, or physiological marker of fatigue taken before and after participation in training, match, or tournament play.

Additionally, the included studies involved all ages and playing standards, sexes (i.e., male, female or intersex) and genders (i.e., men, women, non-binary and other genders).

The exclusion criteria for the studies were as follows:

1) Investigated indoor sports played on grass, ice, or synthetic turf surfaces or individual and doubles sport (e.g., tennis);
2) Involved athletes of non-indoor team sports within the same experimental group as the indoor athletes;
3) Involved athletes with disabilities, returning from injuries, coaching staff or match officials;
4) Included only metabolic markers such as blood lactate and glucose. This is due to ongoing debate in the literature that lactate accumulation may reflect metabolic responses rather than directly contributing to fatigue itself [46,47] while blood glucose levels are more related to prolonged endurance fatigue rather than team sports [48].
5) Investigated fatigue longitudinally (e.g., over an entire mesocycle), or there was greater than 48-hours between the baseline reference measure and the subsequent fatigue measure;
6) Involved other activities (e.g., gym training, cycling, plyometric only sessions.) or match simulation exercise protocols (e.g., treadmill running protocols) between the baseline measurement and post-sports measurement;
7) Investigated a recovery intervention (e.g., the effect of cold-water emersion on recovery) or ergogenic aid (e.g., caffeine supplementation and performance);
8) Articles without an English version available, grey literature (e.g., Thesis), abstract only or conference presentations.

### 2.3. Quality assessment

The methodological quality of the included studies was assessed using the quantitative assessment tool ‘QualSyst’ by Kmet et al. [49]. QualSyst contains 14 items which are scored based on the degree to which each item was met (yes =  2, partial =  1, no =  0). Items not applicable to a particular study design (n =  3) were marked ‘NA’ and excluded from the calculation of the summary score leaving a total of 11 items. A summary score was calculated for each article by summing the total score obtained across relevant items and dividing it by the total possible score. Two of the authors (AC and LP) independently assessed the quality of each paper, after which the any disagreements between quality scores were resolved by discussion between the two authors [49]. A score of > 75% indicated *strong* quality, a score of 55–75% indicated *moderate* quality, and a score < 55% indicated *weak* quality [38,49].

### 2.4. Data extraction

Data were extracted into a custom designed spreadsheet. The characteristics of the subjects (i.e., age, height, weight, sex and playing level) were extracted. The type of sports participation (i.e., training, match play, tournament, or mixed), participation characteristics (e.g., length and number of matches), and fatigue marker measurement schedule (e.g., immediately post-sporting participation, 24-hours post) were also extracted. The fatigue markers were grouped and categorised in accordance with previous reviews on the fatigue response to soccer match-play [41,42]. Fatigue markers were first divided into objective and perceptual markers. The objective markers were further divided into physical and physiological markers. The physical markers consisted of markers measuring physical qualities (e.g., CMJ, mid-thigh pull) and technical skills (i.e., sports specific skills tests). The physiological markers were categorised into markers of oxidative stress, muscle damage, immunology and inflammation, heart rate variability (HRV), and endocrine function. Statistically significant changes to fatigue markers (P <  0.05) and effects sizes were extracted when provided.

### 2.5. Data synthesis

A meta-analysis was not performed as study designs were heterogeneous thus not able to be pooled. The number of papers using each fatigue variable was recorded to determine the most commonly used measures. Change in score from baseline to post-sporting participation was extracted and converted to percentage changes for fatigue markers examined in five or more studies across each fatigue category. When change in score was not available these were manually calculated. Perceptual markers were not converted into percentage change scores as these markers were typically measured on ordinal or interval scales. The percentage change scores were used to calculate ranges for fatigue marker alteration post-sporting participation and to develop the figures presented in the results section.

## 3. Results

### 3.1. Study selection

**Fig 1:**
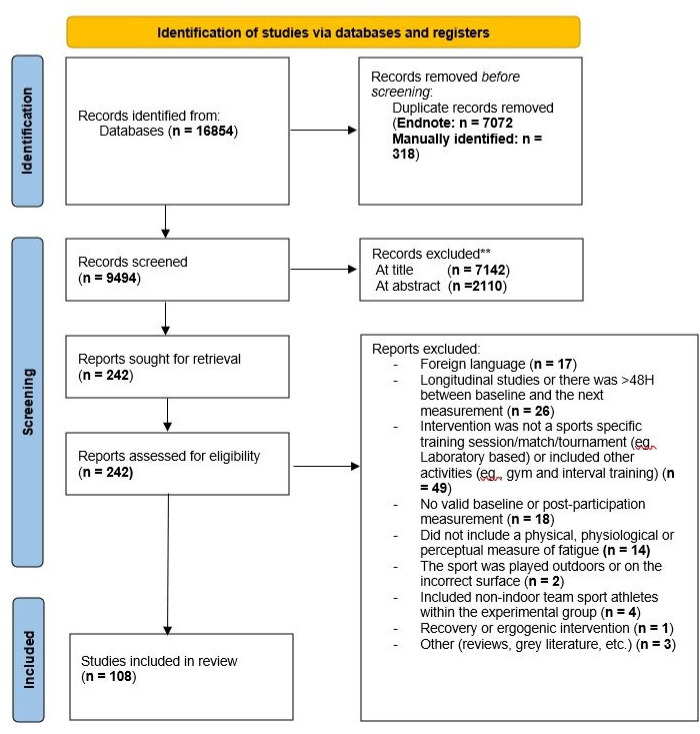
Flow of selection process for the inclusion of eligible studies.

A total of 16,854 articles were identified and retrieved from the database search. Following the removal of duplicates and screening for eligibility, 108 articles remained for inclusion. Fig 1 provides a schematic representation of the PRISMA screening process.

### 3.2. Study quality

Study quality assessment found an average quality score of 86 ±  13% for all included studies. A total of 94 studies were found to have *strong* quality, 10 *moderate* quality [50–59], and four *weak* quality [60–63].The study quality ratings for each study are shown in S1 File.

### 3.3. Study characteristics

Table 2 shows the number of studies assessing each sporting format (i.e., match, training, tournament, mixed) and category of fatigue markers (i.e., physical, physiological, and perceptual). Matches [50,51,54,55,60,64–119] were the most assessed sporting format (n =  61, 57%), followed by training (n =  23, 21%) [53,56,58,59,62,120–137] and tournaments (n =  12, 11%) [52,138–148], while 12 studies (11%) included a combination of matches or tournaments and training [57,61,63,149–157]. Basketball was the most frequently examined sport (n =  45), followed by volleyball (n =  25), handball (n =  20), futsal (n =  16,), netball (n =  8) and floorball (n =  1). Five studies investigate multiple sports [85,103,105,132,153]. The characteristics of studies included in the review are shown in S1 File.

**Table 2:** The number of studies assessing each sporting participation and fatigue measure category for each sport.

	Sports					
	Basketball	Volleyball	Handball	Futsal	Netball	Floorball
**Sport participation**						
Match	27	11	13	10	3	1
Training	8	7	4	3	2	0
Tournament	3	3	1	3	2	0
Mixed (e.g., match and training)	7	4	2	0	1	0
**Fatigue marker category**						
**Physical**						
Physical qualities	20	3	4	5	3	0
Technical	0	0	0	1	0	0
**Physiological**						
Oxidative stress	3	2	3	2	0	0
Muscle damage	10	7	5	8	2	1
Endocrine	15	16	10	5	6	0
Immunology and inflammatory	7	8	8	6	0	1
Hear rate variability	2	1	1	1	0	0
**Perceptual**	11	5	3	5	7	0

Note, some articles assessed multiple sports or multiple categories of fatigue markers.

The majority of studies investigated physiological markers of fatigue (n =  79, 73%), followed by physical (n =  35, 32%) and perceptual (n =  29, 27%). Of the physiological markers, endocrine markers were the most assessed (n =  48, 44%), followed by muscle damage (n =  31, 29%), immunology and inflammation markers (n =  28, 26%), and HRV (n =  5, 5%). Fifty studies (46%) investigate multiple categories of fatigue markers.

### 3.4. Markers of fatigue

**Fig 2:**
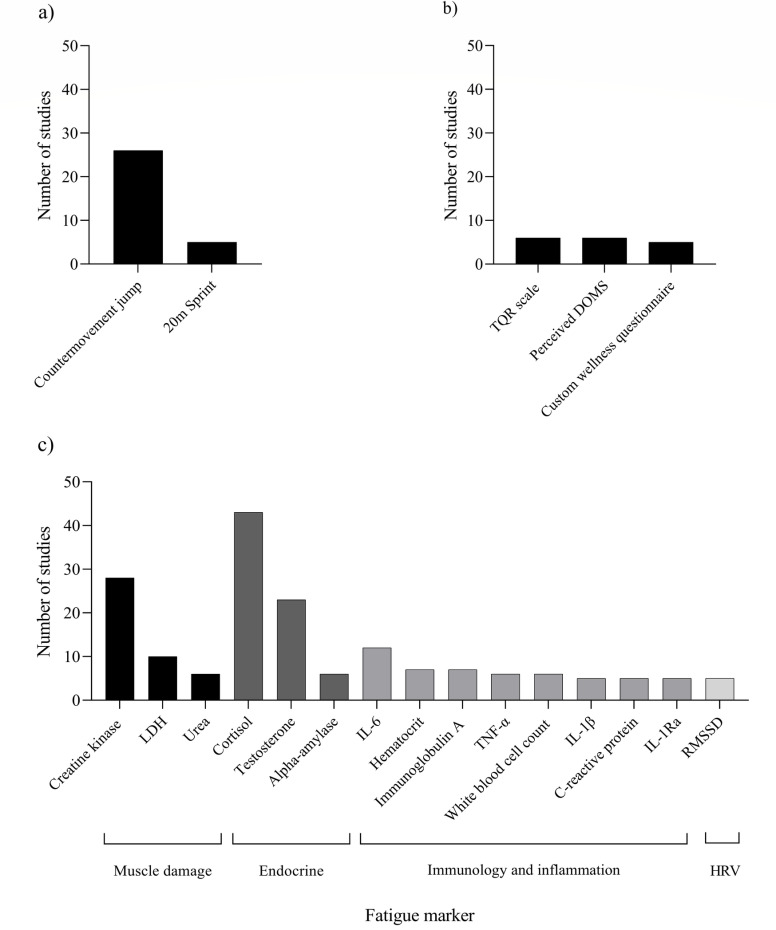
Fatigue markers that have been used in ≥  5 studies for physical markers a), perceptual markers b) and physiological markers c). DOMS, delayed-onset muscle soreness; HRV, heart rate variability; IL-6, interleukin-6; IL-1β, interleukin-1β; IL-1Ra, interleukin-1 receptor agonist; LDH, lactate dehydrogenase; RMSSD, Root mean square of the successive differences of R-R intervals; TNF-α, tumour necrosis factor- α; TQR, total quality recovery.

The number of studies examining common markers of fatigue (i.e., examined by five or more studies) are shown in Fig 2. A full breakdown of fatigue markers, and the number of studies examining them, are shown in S1 File.

#### 3.4.1. Physical markers.

A total of 33 different physical markers were used to assess fatigue in the athletes (S1 File). The CMJ was the most frequently examined marker of physical fatigue (n =  26) [51,56,74,77,79,91,101,107,110,117–120,125,127,129,131,134,137,139,140,142,148,149,151,156], however, 33 different CMJ variables were examined across these studies (S1 File). Countermovement jump height was examined by all of these studies, but was calculated via three different methods: flight time (n = 16, 62%) [51,74,77,79,91,101,107,110,117–119,125,137,139,142,149], take-off velocity (n = 9, 35%) [56,120,127,129,131,134,148,151,156], and video camera with body marker analysis (n = 1, 4%) [140].

Multiple testing methods and markers were used to determine other physical qualities associated with fatigue (S1 File), these include: linear speed (number of markers =  3), change of direction ability (number of markers =  5), muscular strength (number of markers =  6) and repeated sprint ability (number of markers =  4). Only one paper examined a technical marker of fatigue (finishing kick performance in futsal) (80).

#### 3.4.2. Physiological markers.

Ninety-three physiological markers to assess fatigue were identified in the studies included in the review, shown in S1 File. A total of 13 different endocrine markers were examined across the physiological marker studies. The most frequently assessed endocrine markers were cortisol (n =  43) followed by testosterone (n = 23) (S1 File).

Muscle damage was examined using eight different markers with [CK] (n =  28) and lactate dehydrogenase (LDH) (n =  10) the most frequently examined. Interleukin-6 was the most examined inflammatory and immune marker of fatigue (n =  12), with all other immune and inflammatory markers used in less than eight studies (S1 File). The most frequently assessed HRV marker was the root mean square of the successive differences of R-R intervals (RMSSD) (n =  5) with 12 different HRV markers utilised in total (S1 File). Despite 30 different markers of oxidative stress being used across the included studies, the most frequently examined marker was protein carbonyls which appeared in four studies.

#### 3.4.3. Perceptual markers.

There were 16 different perceptual tools used as markers of fatigue across the included studies (S1 File). The most frequently used tools were the total quality of recovery scale (TQR) (n =  6) and perceived delayed onset muscle soreness (DOMS) (n =  6), followed by custom wellness questionnaires (n =  5). Mood state was assessed in nine studies which utilised the adapted brief assessment of mood (BAM+) (n =  3), Brunel mood scale (BRUMS) (n =  4), profile of mood states (POMS) (n =  1) and Hooper index (n =  1).

### 3.5. The acute fatigue response to sports participation

#### 3.5.1. Physical response.

**Fig 3:**
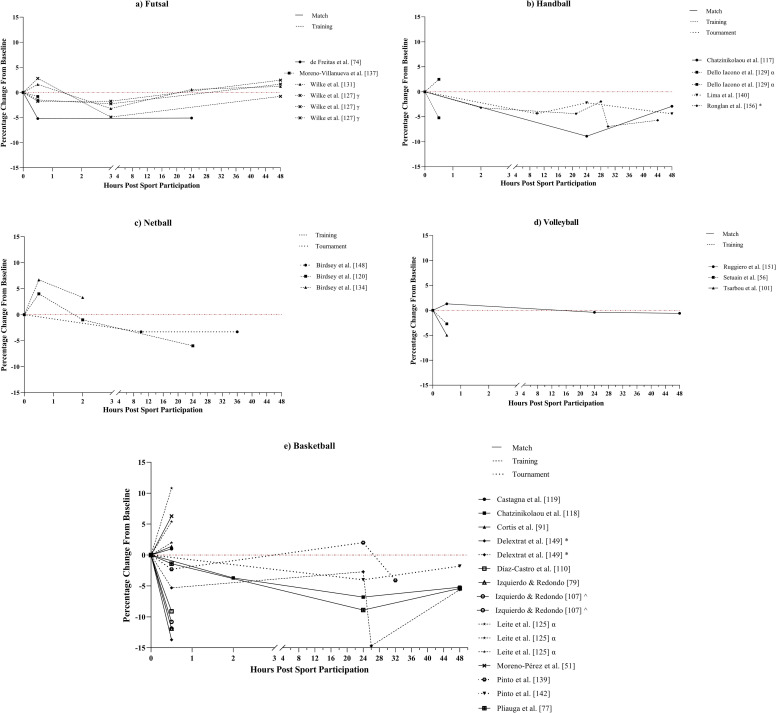
Percentage changes from baseline post-sporting participation in countermovement jump (CMJ) height in studies assessing a) Futsal, b) Handball, c) Netball, d) Volleyball, and e) Basketball athletes. Each line represents a sporting population from a study. The line pattern represents sporting format, i.e. Match, training, or tournament. #: Study examining multiple sports, ~ : Study examining male and female athletes, * : Study examining multiple sporting formats, ^: Study examining multiple age groups, α: Study examining multiple training or match conditions (e.g., different small sided games), β: Case study, γ: Study examining athletes with differing recovery profiles (e.g., slow perceptual recovery, slow physical recovery, etc.).

Countermovement jump height response (Fig 3) varied immediately post-sporting participation (i.e., ≤  1-hour); 10 studies demonstrated a decrease in jump height [56,74,77,79,101,107,110,137,139,149], eight an increase [51,91,119,120,125,131,134,151], and two both a decrease and an increase [127,129] with CMJ height alterations ranging from -13.7% to 10.8%. Jump height response over the following hours (up to 24-hours post-exercise) was similar across all sports, either decreasing back to or below baseline levels, or remaining depressed relative to baseline. Fourteen studies measured CMJ height at ≥  24-hours post-sport participation [74,77,117,118,120,127,131,139,140,142,148,149,151,156], of which eight experienced peak CMJ height disturbances within 24-hours. However, five studies observed a continued decrease past 24-hours, all of which were training or tournament studies with repeated exposures to sporting participation [139,140,148,149,156].

Linear speed and repeated sprint performance were measured to assess fatigue in basketball and handball athletes only. Linear speed was significantly reduced post-sports participation (P <  0.05) in all nine studies testing for it, regardless of the test used or sport participation format [77,79,91,107,117,118,139,149,156]. The change in 20-metres sprint performance ranged from 3.1% to 4.2% immediately post-sporting participation [79,107,139,149,156]. Similarly, repeated sprint ability was found to significantly (P <  0.05) reduce by in all studies examining it [65,87,117–119,124,128] with changes to the basketball line drill test ranging from 2.0% to 5.6% immediately post-exercise [117–119,128]. Muscular strength and change of direction ability were only examined across basketball, handball, and futsal. Strength performance was found to significantly decrease (P <  0.05) across six studies (n =  6) [80,117,118,137,149,156] but remained unchanged in two studies [90,91]. Bench press (the most examined strength test) performance changes ranged from -6.5% to 0.2% immediately post basketball matches and -6.7% below baseline 24-hours post a handball match [90,117,118]. Change of direction ability across these sports was unaltered in four studies [90,119,128,131] but significantly decreased in three (P <  0.05) [117,118,127].

#### 3.5.2. Physiological response.

**Fig 4:**
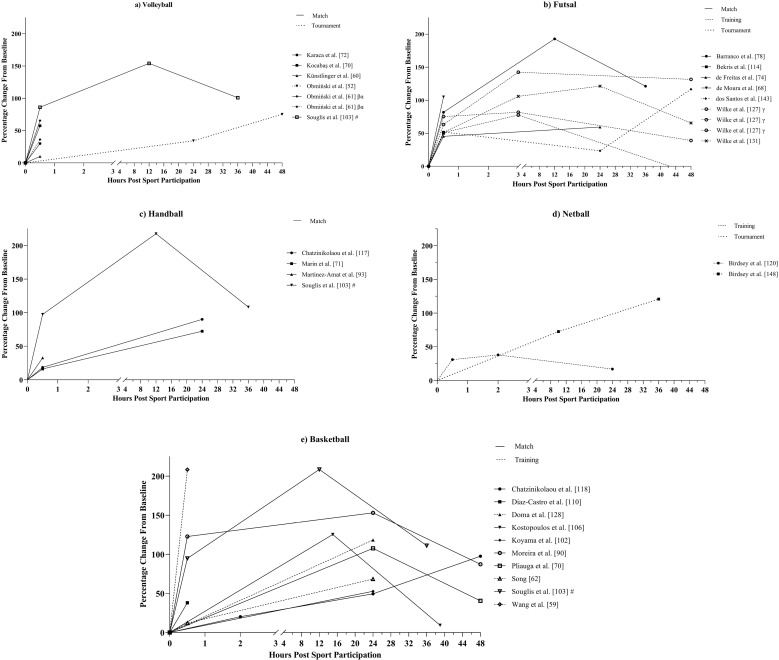
Percentage changes from baseline post-sporting participation of creatine kinase in studies assessing a) Volleyball, b) Futsal, c) Handball, d) Netball, and e) Basketball athletes. Each line represents a sporting population from a study. The line pattern represents sporting format, i.e. Match, training, or tournament. #: Study examining multiple sports, ~ : Study examining male and female athletes, * : Study examining multiple sporting formats, ^: Study examining multiple age groups, α: Study examining multiple training or match conditions (e.g., different small sided games), β: Case study, γ: Study examining athletes with differing recovery profiles (e.g., slow perceptual recovery, slow physical recovery, etc.).

Changes in [CK] (Fig 4) observed in studies included in this review were similar across all sports, increasing immediately post-exercise (i.e., ≤  1-hour), regardless of sporting participation format [59–62,68,70–72,74,78,90,93,103,110,114,117,120,127,131,143]. However, the degree of increase varied across the studies and ranged from 10.0% to 208.4%. Creatine kinase concentration continued to increase up to 24-hours post-sporting participation with change from baseline ranging from 17.0% to 153.1% at 24-hours. Only 11 studies measured [CK] past 24-hours post-exercise [52,77,78,90,103,106,118,127,131,143,148] and seven measured up to 48-hours post-exercise [52,77,90,118,127,131,143]. Following 24-hours, [CK] decreased from peak values in seven studies, however, only one of these demonstrated a group of athletes returning to baseline values by their final measurement [127]. Four studies demonstrated a continued increase in [CK] past 24-hours (range 75.3% to 120.8%), of which three were tournaments with repeated exposures to sport between measurements [52,143,148].

**Fig 5:**
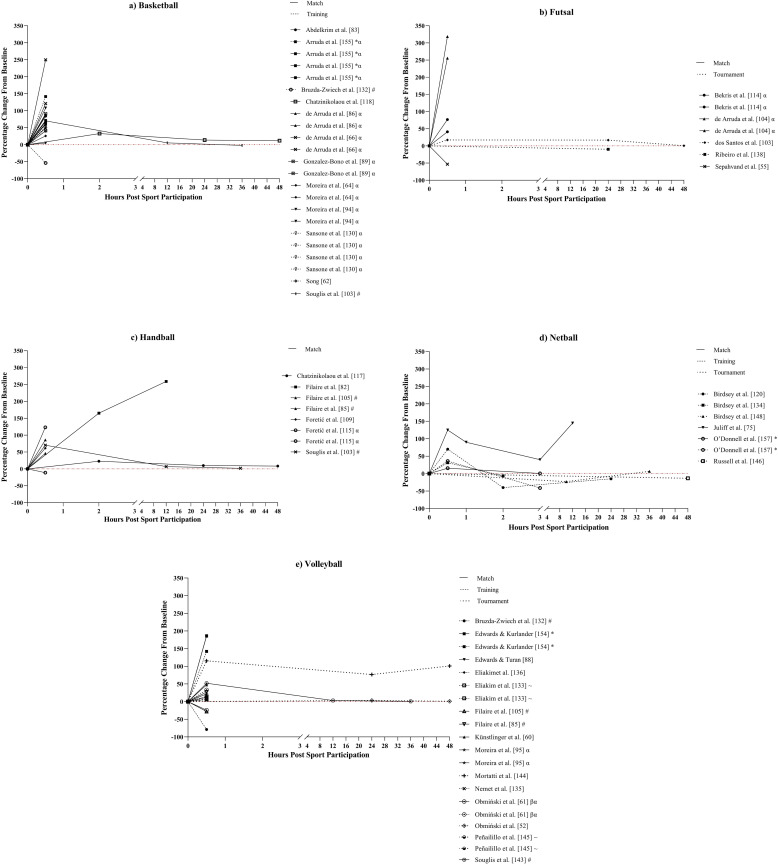
Percentage changes from baseline post-sporting participation of cortisol in studies assessing a) Basketball, b) Futsal, c) Handball, d) Netball, and e) Volleyball athletes. Each line represents a sporting population from a study. The line pattern represents sporting format, i.e. Match, training, or tournament. #: Study examining multiple sports, ~ : Study examining male and female athletes, * : Study examining multiple sporting formats, ^: Study examining multiple age groups, α: Study examining multiple training or match conditions (e.g., different small sided games), β: Case study, γ: Study examining athletes with differing recovery profiles (e.g., slow perceptual recovery, slow physical recovery, etc.).

The majority of studies that examined cortisol only measured cortisol immediately post-sporting participation (i.e., ≤  1-hour), with 14 studies measuring cortisol at time points greater than 1-hour post-exercise across the 43 studies examining cortisol response (Fig 5) [52,75,82,103,117,118,120,134,138,143,144,146,148,157]. The immediate post-exercise cortisol response was similar between all sports with cortisol increasing in 31 studies (range 4.8% to 318.8%) [60–62,64,66,75,83,85,86,88,89,94,95,103–105,109,114,115,120,130,133–136,143–145,154,155,157]. Following 1-hour post-exercise, cortisol levels reduced towards baseline levels or below in 11 studies across all sports [52,103,117,118,120,134,138,143,146,148,157] but remained elevated in three studies [75,82,144].

**Fig 6:**
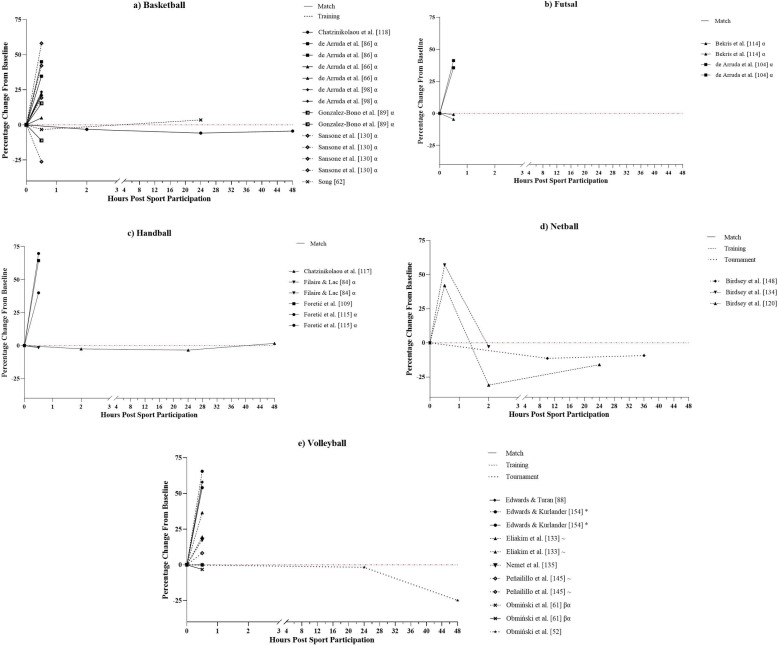
Percentage changes from baseline post-sporting participation of testosterone in studies assessing a) Basketball, b) Futsal, c) Handball, d) Netball, and e) Volleyball athletes. Each line represents a sporting population from a study. The line pattern represents sporting format, i.e. Match, training, or tournament. #: Study examining multiple sports, ~ : Study examining male and female athletes, * : Study examining multiple sporting formats, ^: Study examining multiple age groups, α: Study examining multiple training or match conditions (e.g., different small sided games), β: Case study, γ: Study examining athletes with differing recovery profiles (e.g., slow perceptual recovery, slow physical recovery, etc.).

Testosterone response (Fig 6) was similar between all sports. Testosterone increased immediately post-exercise in the majority of studies (n =  13, range 5.0% to 69.9%) [66,86,88,98,104,109,115,120,133–135,145,154], and decreased or remained around baseline levels in three studies (range -4.6% to -0.9%) [62,84,114]. A further three studies demonstrated both increased and decreased testosterone in their subsets of athletes immediately post-exercise [61,89,130]. Testosterone was only assessed after >  1-hour post-exercise in seven studies. From 2-hours onwards testosterone decreased to baseline levels or below in all netball studies (range −2.9% to −31%) [120,134,148], decreased to 24.9% below baseline levels at 48-hours in a single volleyball case study [52], and remained close to baseline levels in handball and basketball (range −5.8% to 3.5%) [62,117,118].

Of studies that examined markers of oxidative stress, eight demonstrated significantly increased levels of oxidative stress post-exercise in basketball (P <  0.05) [53,54,118], volleyball [70,72], futsal [74], and handball [71,117]. Regarding immunology and inflammatory function, ten studies examining inflammatory cytokine response found that serum levels significantly increased immediately post-sport participation (P <  0.05) [68,71,74,103,117,118,129,133,135,136] with increases in interleukin-6 ranging from 24.5% to 400.0%. One study found a decrease in interleukin-6 of 46.8% post-handball training [126]. White blood cell count was found to significantly increase (P <  0.05) immediately post-sport participation in six studies [72,106,117,118,123,126] with increases ranging from 10.4% to 35.5%. Salivary immunoglobulin-A response was mixed across studies with two demonstrating a significant increase (P < 0.05) [57,132], two a significant decrease (P <  0.05) [67,73], and two reporting no change to immunoglobulin-A levels [94,108]. Studies examining HRV demonstrated mixed results with three reporting no alterations to HRV markers [116,138,147] and two a decrease in HRV [111,121].

#### 3.5.3. Perceptual responses.

Tools to assess mood state (BAM+ , BRUMS, POMS, and the Hooper index) were utilised in netball, basketball, and futsal athletes only. Of these studies, eight found significant negative disturbances (P <  0.05) to mood post-sports participation with subscales of fatigue, vigour, anger, depression, and soreness being affected [89,120,127,131,139,141,142,148]. In netball overall mood was negatively altered at 2-hours post a netball specific training session but had recovered by 24-hours post [120], however, when repeated exposures over the course of a tournament were examined overall mood state continued to decrease as time progressed [148]. This progressive decline in mood state was not consistent across futsal and basketball tournament studies, with two studies showing no cumulative effect of match participation on mood state subscales [141,142]. Perceived recovery tools (TQR and short recovery stress scale) were significantly negatively impacted for up to 12-hours after match or training participation regardless of sport, but returned to baseline levels by 24-hours post-exercise [92,127,152]. However, all tournament studies found a significant decline in perceived recovery over the course of the tournament in basketball and netball athletes [131,139,142]. Perceived DOMS was also found to significantly increase in all studies (n =  6) [74,90,117,118,128,140] with disturbances still present at 48-hours post-sporting participation in all studies.

## 4. Discussion

This systematic review aimed to identify the methods and markers used to determine fatigue in indoor court-based team sports and to describe the acute fatigue response to sport participation (i.e., match play, sports-specific training, and tournaments) experienced by athletes in each sport. A total of 108 studies were included in the final review with basketball being the most researched sport (n =  45, 42%) and matches the most researched sporting format (n =  61, 56%). The study quality criteria assessment determined that 87% (n =  94) of studies exhibited *strong* quality, 9% (n =  10) *moderate* quality, and 4% (n =  4) *weak* quality (S1 File). The primary findings of this review are, i) the most common markers used to assess acute fatigue in indoor court-based team sports are cortisol (n =  43, 40%), [CK] (n =  28; 26%), CMJ height (n =  26, 24%), and testosterone (n =  23, 21%) (Fig 3), ii) the ability to compare trends in the acute fatigue response between sports and match or training responses was impacted by the large variability in both the markers and methods to determine fatigue and timeline of marker measurement across the studies, and iii) [CK] demonstrated the most consistent response among fatigue markers across the 48-hour period post-sporting participation (Fig 4), increasing between 10% and 208% from baseline levels and peaking at approximately 24-hours post-sport. However, the large range in increases for [CK] and the high interindividual variability of this marker suggest it is best used in combination with physical and perceptual markers of fatigue such as the CMJ and perceived recovery or DOMS.

### 4.1. Methods and markers used to determine fatigue

Across the studies included in the review a large range of markers were used to assess fatigue with a total of 142 different fatigue markers examined across 108 studies. The number of fatigue markers observed is more than a recent systematic review on post-match recovery in team ball sports which found 59 different physical and biochemical markers of fatigue across 28 studies [39]. However, this review did not include perceptual markers of fatigue and markers of HRV and did not examine the fatigue response of training in addition to match-play. Physiological fatigue markers were the most common throughout the studies in the present review with 73% (n =  79) of studies examining a total of 93 different markers (S1 File). This is considerably more than physical fatigue markers (S1 File) which were examined in 32% (n =  35) of studies, using 33 different markers. Sixteen perceptual markers (S1 File) were included across 27% (n =  29) of studies. The greater use of physiological markers demonstrated in the present study contrasts with the markers reportedly utilised by coaches and teams in practical settings. A recent systematic review found that biochemical markers were the utilised less by sports teams when compared with physical tests or self-reported measures [158]. That review reported as low as 4% of coaches utilise endocrine monitoring [158], while the present review found that endocrine markers where the most examined fatigue marker category in research (n =  46). This finding is aligned with recent research suggesting that there is a gap between the evidence base and applied practices [159]. Many teams lack the resources, equipment, staff expertise and knowledge base to incorporate costly, time-inefficient, monitoring protocols that frequently require access to a laboratory setting and as such prefer to utilise physical or subjective monitoring tools that are easy to implement and are cost effective [160,161]. It may be argued that research into the physiological markers of fatigue may help further the understanding as to how fatigue develops within a given sport. However, there is currently no consensus on a definitive physiological marker of fatigue [162], due in part to the high inter-individual variability and large reference ranges of physiological markers in athletic populations [162,163]. This may explain why such a large range of physiological markers are currently being used in research and why practitioners may be hesitant to employ them in the field. Future research should aim to examine what fatigue markers are being utilised in the field and determine which of these are most sensitive to detecting acute fatigue in athletes.

As a result of the large range of fatigue markers, 121 out of 142 markers were examined in less than five studies each. This limited the ability to determine trends in the fatigue response between sports. Despite this, there were several markers that were frequently utilised across the included studies with cortisol, [CK], the CMJ, and testosterone all being examined in ≥  23 (21%) studies. The most examined marker of physical fatigue was the CMJ, with CMJ height being the most commonly used variable, utilised in all 26 studies examining CMJ performance (Fig 3). This is in line with a previous review on post-match recovery in team ball sports [39] which found CMJ height to be the most commonly used physical marker, utilised in 43% of studies in that review. However, the methods used to calculate CMJ height across the present review varied, with studies calculating jump height via the flight-time (n =  16), take-off velocity (n =  9) and video analysis (n =  1) methods. Despite these methods all displaying high reliability with coefficients of variation (CV) of <  6% [164–167], the sensitivity of jump height alone to detect fatigue has been questioned due to athletes altering their jump strategy by taking longer to produce the required force to maintain jump height [168]. Consequently, some research recommends including time-related CMJ variables that reflect jump strategy (e.g., eccentric duration, total duration, flight time to contact time ratio) in combination with jump outcome variables (e.g., jump height, peak power, peak force) when analysing fatigue related jump alterations [168–170]. Only a single study in this review included time-related CMJ variables [151], thus, future research utilising the CMJ to assess fatigue in indoor court-based team sports may be improved by including variables that reflect jump strategy. Amongst the multitude of physiological fatigue markers, the endocrine markers cortisol (n = 43) and testosterone (n =  23), and muscle damage marker [CK] (n =  28) emerged as the most frequently examined. Cortisol is commonly linked to increased physiological stress and protein degradation [171,172], while testosterone is an androgenic-anabolic hormone associated with increased protein synthesis and reduced protein catabolism [173]. Consequently, the ratio of testosterone to cortisol (T:C) has been proposed to represent the ratio of anabolic to catabolic activity within athletes and may be more appropriate than examining the hormones in isolation in sporting settings [174]. However, only four studies within the present review examined the T:C ratio, potentially missing a key interaction within the post-exercise endocrine response of athletes which should be considered in future research.

Studies examining the CMJ, [CK], cortisol, and testosterone also displayed large variation in the timeline of marker measurement, further limiting the ability to compare results between studies. Only 42% (n =  11) of studies examining CMJ height (Fig 3) and 39% (n =  11) of studies examining [CK] (Fig 4) measured these markers >  24-hours post-sport participation while cortisol (Fig 5) and testosterone (Fig 6) were measured at timepoints >  1-hour post-sporting participation in only 33% (n =  14) and 30% (n =  7) of studies respectively. With previous reviews on fatigue in team ball sports reporting that disturbances to fatigue measures tend to peak within 24 to 48-hours [39,42], this suggests that a large percentage of studies are missing a substantial window in the fatigue response of athletes. Additionally, endocrine hormones such as cortisol and testosterone are diurnal in nature and are impacted by the time-of-day of their measurement [175], indicating that researchers should include baseline measurements on a time-matched control day to control for this. This is supported by a recent meta-analysis which found that variation between studies in testosterone and cortisol response may be influenced by study design, highlighting the timing of baseline measurements prior to exercise as a contributing factor and calling for standardised hormonal measurements across studies [176]. Hence, it is recommended that future research should ensure that the appropriate timeline of measurement is being used for the selected fatigue markers, considering factors outside of sporting participation that may influence measurement, such as time of day.

### 4.2. Describing the acute fatigue response

Due to the large number of markers examined across studies included in this systematic review and the variability in measurement methods and fatigue response, only the fatigue markers displaying the most consistent trends across the 48-hour period post-sport will be discussed. These markers include [CK], the CMJ and perceptual markers of mood state, recovery, and DOMS.

Creatine kinase concentration displayed the most consistent response over 48-hours across all sports, increasing by up to 208% and peaking at 24-hours post-sporting participation before beginning to decline towards baseline levels [55,56,71,88,91,104,115,131]. This is consistent with a previous review in team ball sports which found 89% of studies demonstrated peak [CK] values within ≤  24-hours [39]. Creatine kinase is an enzyme stored within muscle cells that permeates the cell walls and leaks into the blood stream when muscle damage takes place, thus an increase in [CK] from baseline levels indicates that muscle damage has occurred [177]. It is possible that the high eccentric load on the hard playing surfaces typically experienced by indoor athletes induced muscle damage which led to the increases in [CK] [12–14]. The present review also found that of the four studies that demonstrated a continued increase in [CK] past 24-hours, three were tournaments [52,143,148], suggesting a cumulative muscle damaging effect to repeated exposures of indoor sport when athletes have limited time to recover. However, it should be noted that currently there no defined threshold for [CK] increase that indicates a significant degree of muscle damage has taken place in an individual and that there is a poor temporal relationship between [CK] and physical performance recovery after exercise [148,178,179]. Moreover, although [CK] displayed a consistent pattern in its response, the degree of increases varied greatly between studies (range 10% to 208%) (Fig 4). This is consistent with a review and meta-analysis on fatigue in rugby union which found large variability in the response of biochemical fatigue markers between studies [180]. There is limited research into the reliability and sensitivity of [CK] with previous research in rugby union and rugby league demonstrating CVs of 26% and 27% and a smallest worthwhile change (SWC) of 8.6% [164,179]. Creatine kinase also displays wide reference ranges in athletic populations with interindividual variability being impacted by factors such as gender and age [162,181]. This may explain the large ranges in [CK] increase seen in this review. These factors suggest that while an increase in [CK] may indirectly indicate the presence of muscle damage, the degree of muscle damage within athletes cannot be demonstrated using [CK] alone and that other fatigue markers should be considered in addition to [CK].

Countermovement jump height also displayed consistent responses to sport participation but only from time points >  1-hour post-exercise [74,117,118,120,127,131,142,149], decreasing to baseline levels or below. The variability in results immediately post-sporting participation [51,91,119,120,125,131,134,151] may be due to a number of factors such as the differing fitness levels, playing standards and training age of the athletes, differences in positional demands within sports, differences in training or match participation times of athletes, potential post-activation potentiation effects, and increases in muscle temperature and cellular water content post-exercise [182–184]. The declines in CMJ height following 1-hour are consistent with a previous review [39] and may be linked to the induction of exercise induced muscle damage due the high eccentric load of indoor court sports [9,11,169]. The resulting inflammatory processes, sarcomere disruption, and disturbed ionic flux in the hours following high volume eccentric exercise has previously been associated with reduced force production [169–171] and coincides with the increases in [CK] up to 24-hours post-sport. These findings suggest that combining the use of the CMJ and [CK] to detect the presence of acute muscle damage induced changes in indoor court-based team sports performance may provide a more robust measurement of muscle damage linked fatigue. However, both these measures involve the use of expensive equipment, are potentially invasive, and the maximal nature of the CMJ may not suit certain environments with high congestion schedules [160,185].

Perceptual markers of fatigue may provide a cost effective and easy to implement addition or alternative to the markers above [160,185]. Markers assessing mood state [89,120,127,131,139,141,142,148], perceived recovery [74,115,131,133,139,152], and DOMS [74,90,117,118,128,140] all displayed significant (P <  0.05) negative alterations post-exercise, regardless of sport. Negative alterations to mood state and perceived recovery were present within the 2-hours post-sporting participation but returned to baseline by 24-hours post single training or match exposures while perceived DOMS displayed significant increases up to 48-hours post-sport. Self-reported perceptual markers have previously been reported to be more sensitive to acute changes in training load than objective markers [186] which concurs with the consistent responses across the different tools used to assess perceptual fatigue. Within the present review perceived recovery was also demonstrated to be sensitive to cumulative loads associated with tournaments [131,139,142] and can be easily assessed with tools such as the TQR scale. The evidence in this review suggests that perceptual fatigue markers may be used to assess acute fatigue in indoor court-based team sports, however, they were also the least researched category of fatigue markers (27% of studies) so further research in these sports is warranted.

### 4.3. Limitations

There are some inherent limitations to this review that should be highlighted. Firstly, the inclusion of multiple athlete age groups and playing standards may have impacted the variability of the results. Despite this, as there is limited research into the acute fatigue response of indoor court-based team sport athletes, this review included athletes from youth to senior international standard. Secondly, this review did not consider the impact of the varying external loads experienced by athletes (i.e., time, type, and amount of load exposure) on their fatigue response due to the majority of studies not reporting athlete loading throughout the literature. Future research on the acute fatigue response should quantify and report both the internal and external loads athletes are exposed to during sport participation. Thirdly, all measurements ≤  1-hour post-sport were treated as immediately post-sport as multiply studies did not precisely report the time of measurement within this timeframe (e.g., 15-minutes or 30-minutes) and there may have been differences in marker responses within these timeframes. Furthermore, this review did not consider the impact of day of training relative to match day (e.g., match day minus one versus minus two) as this data was not reported in the majority of studies. Future research should aim to compare the fatigue response on different training days relative to match day. Lastly, despite the inclusion of all sexes and genders, the differences in acute fatigue response between male and female athletes was not considered as there was a disparity in the amount of research conducted on female compared to male athletes with only 36% of studies including female athletes. Future research should address this disparity by examining fatigue in female indoor court-based team sports.

## 5. Conclusion

This study systematically reviewed the literature on the acute fatigue response of indoor court-based team sports. Physiological markers were the most examined fatigue marker category in the literature, however, this contrasted with previous reports that state these markers are rarely utilised in practical sports settings. There was large variability in the markers and methods used to determine fatigue which limited the ability to determine trends in the fatigue response of athletes and compare the fatigue response between different sports and training modalities. Additionally, it was found that studies examining common fatigue markers such as the CMJ, [CK], cortisol, and testosterone could be improved by carefully selecting the appropriate combination of markers to assess a given quality and ensuring a long enough timescale of measurement to fully capture the fatigue response of athletes. Creatine kinase displayed the most consistent trend out of all the markers, increasing post-sport in all studies examining it. However, the large range in increases for [CK], low reliability and high interindividual variability of measurement suggest it is best used in combination with physical and perceptual markers of fatigue. Future researchers should carefully consider their choice of fatigue markers focusing on markers that are transferable to practitioners in the field and display high levels of reliability. Further research is also still required to determine which markers or combination of markers bests describe a given fatigue response.

## Supporting information

S1 File Supplementary tables. (DOCX)

S2 File List of studies identified in literature search. (CSV)

S3 File Data extracted from included studies. (XLSX)

S1 Checklist PRISMA. (DOCX)
