# Supplementary material for: Acute fatigue in indoor court-based team sports: A systematic review
Source: PLoS One. 2025 Feb 14;20(2):e0316831. doi: 10.1371/journal.pone.0316831 (PMC11828399; doi:10.1371/journal.pone.0316831)
Supplement: S1 File — (DOCX) [file pone.0316831.s001.docx]

| **S1: Supplementary Table 1. Quality assessment ‘Qualsyst’ scores** | | | | | | | | | | | | | |
| --- | --- | --- | --- | --- | --- | --- | --- | --- | --- | --- | --- | --- | --- |
| Authors | Question sufficiently described | Study design evident and appropriate | Method of subject group selection or source of variables described and appropriate | Subject characteristics sufficiently described | Outcome and exposure measures well defined and robust to measurement bias | Sample size appropriate | Analytic methods described and appropriate | Some estimate of variance is reported for the main results? | Controlled for confounding | Results reported in sufficient detail | Conclusion supported by the results | Quality Score | Rating |
| Abdelkrim et al. [83] | 2 | 2 | 2 | 2 | 2 | 2 | 2 | 2 | 2 | 2 | 1 | 95 | Strong |
| Akhundov et al. [97] | 2 | 2 | 2 | 2 | 2 | 2 | 2 | 2 | 1 | 2 | 2 | 95 | Strong |
| Amin et al. [126] | 2 | 2 | 2 | 2 | 2 | 2 | 2 | 2 | 1 | 2 | 2 | 95 | Strong |
| Arruda et al. [155] | 2 | 2 | 2 | 2 | 1 | 2 | 2 | 2 | 2 | 2 | 1 | 91 | Strong |
| Barranco et al. [78] | 2 | 2 | 2 | 2 | 2 | 1 | 2 | 2 | 1 | 2 | 2 | 91 | Strong |
| Bekris et al. [114] | 2 | 2 | 2 | 2 | 2 | 2 | 1 | 2 | 1 | 2 | 2 | 91 | Strong |
| Biondi et al. [123] | 2 | 2 | 2 | 1 | 2 | 1 | 1 | 2 | 1 | 2 | 2 | 82 | Strong |
| Birdsey et al. [148] | 2 | 2 | 2 | 2 | 2 | 1 | 2 | 2 | 1 | 2 | 2 | 91 | Strong |
| Birdsey et al. [120] | 2 | 2 | 2 | 2 | 2 | 1 | 2 | 2 | 2 | 2 | 2 | 95 | Strong |
| Birdsey et al. [134] | 2 | 2 | 2 | 2 | 2 | 1 | 2 | 2 | 1 | 1 | 2 | 86 | Strong |
| Brudza-Zwiech et al. [132] | 2 | 2 | 2 | 1 | 2 | 2 | 2 | 2 | 1 | 2 | 2 | 91 | Strong |
| Caetano Júnior et al. [108] | 2 | 2 | 2 | 2 | 1 | 2 | 2 | 2 | 1 | 1 | 2 | 86 | Strong |
| Caprino et al. [87] | 2 | 1 | 2 | 2 | 2 | 1 | 2 | 2 | 1 | 2 | 2 | 86 | Strong |
| Castagna et al. [119] | 2 | 2 | 2 | 2 | 2 | 2 | 2 | 2 | 1 | 2 | 2 | 95 | Strong |
| Charlot et al. [141] | 2 | 2 | 2 | 2 | 1 | 1 | 2 | 2 | 1 | 2 | 2 | 86 | Strong |
| Chatzinikolaou et al. [118] | 2 | 2 | 2 | 1 | 2 | 2 | 2 | 2 | 2 | 2 | 2 | 95 | Strong |
| Chatzinikolaou et al. [117] | 2 | 2 | 2 | 2 | 2 | 2 | 2 | 2 | 1 | 2 | 2 | 95 | Strong |
| Chavez et al. [50] | 2 | 1 | 2 | 2 | 2 | 0 | 1 | 1 | 0 | 2 | 1 | 64 | Moderate |
| Conte et al. [113] | 2 | 2 | 2 | 2 | 2 | 1 | 2 | 2 | 1 | 2 | 2 | 91 | Strong |
| Cortis et al. [91] | 2 | 2 | 2 | 2 | 2 | 1 | 2 | 2 | 1 | 2 | 2 | 91 | Strong |
| Cury-Boaventura et al. [76] | 1 | 2 | 2 | 2 | 2 | 2 | 2 | 2 | 1 | 2 | 2 | 91 | Strong |
| de Arruda et al. [86] | 1 | 1 | 2 | 2 | 2 | 2 | 2 | 2 | 1 | 2 | 1 | 82 | Strong |
| de Arruda et al. [104] | 2 | 1 | 2 | 2 | 1 | 2 | 2 | 2 | 1 | 2 | 1 | 82 | Strong |
| de Arruda et al. [66] | 2 | 2 | 2 | 2 | 2 | 1 | 2 | 2 | 1 | 2 | 2 | 91 | Strong |
| de Arruda et al. [98] | 2 | 1 | 2 | 1 | 2 | 1 | 2 | 2 | 1 | 2 | 2 | 82 | Strong |
| de Freitas et al. [74] | 2 | 2 | 2 | 2 | 2 | 1 | 2 | 2 | 1 | 2 | 2 | 91 | Strong |
| de Moura et al. [96] | 2 | 2 | 2 | 2 | 1 | 2 | 2 | 2 | 1 | 2 | 1 | 86 | Strong |
| de Moura et al. [68] | 1 | 2 | 2 | 2 | 2 | 2 | 1 | 2 | 1 | 2 | 2 | 86 | Strong |
| Delextrat et al. [149] | 2 | 2 | 2 | 2 | 2 | 1 | 2 | 2 | 1 | 2 | 2 | 91 | Strong |
| Delextrat et al. [65] | 2 | 2 | 2 | 2 | 2 | 1 | 2 | 2 | 1 | 2 | 2 | 91 | Strong |
| Dello Iacono et al. [129] | 2 | 2 | 2 | 2 | 2 | 1 | 2 | 2 | 2 | 2 | 2 | 95 | Strong |
| Díaz-Castro et al. [110] | 2 | 2 | 2 | 2 | 2 | 2 | 2 | 2 | 1 | 2 | 2 | 95 | Strong |
| Djordjevic et al. [122] | 2 | 2 | 2 | 2 | 2 | 2 | 2 | 2 | 2 | 2 | 2 | 100 | Strong |
| Doma et al. [128] | 2 | 2 | 2 | 2 | 2 | 2 | 2 | 2 | 1 | 2 | 2 | 95 | Strong |
| dos Santos et al. [143] | 1 | 2 | 2 | 2 | 2 | 2 | 2 | 2 | 1 | 2 | 1 | 86 | Strong |
| Edmonds et al. [116] | 2 | 1 | 2 | 2 | 1 | 2 | 2 | 2 | 1 | 2 | 2 | 86 | Strong |
| Edwards & Kurlander [154] | 1 | 2 | 1 | 2 | 2 | 2 | 1 | 2 | 1 | 2 | 1 | 77 | Strong |
| Edwards & Turan [88] | 1 | 2 | 2 | 2 | 2 | 2 | 2 | 2 | 1 | 2 | 2 | 91 | Strong |
| Eliakim et al. [133] | 2 | 2 | 2 | 2 | 2 | 2 | 1 | 2 | 1 | 2 | 1 | 86 | Strong |
| Eliakim et al. [136] | 2 | 2 | 2 | 2 | 2 | 2 | 2 | 2 | 1 | 1 | 2 | 91 | Strong |
| Fernández-Rio et al. [153] | 2 | 1 | 1 | 2 | 1 | 2 | 2 | 2 | 1 | 2 | 1 | 77 | Strong |
| Fien et al. [112] | 1 | 2 | 2 | 2 | 2 | 1 | 2 | 2 | 1 | 2 | 2 | 86 | Strong |
| Filaire et al. [82] | 1 | 2 | 2 | 2 | 2 | 2 | 1 | 2 | 1 | 2 | 1 | 82 | Strong |
| Filaire et al. [105] | 2 | 2 | 2 | 2 | 2 | 2 | 1 | 2 | 1 | 1 | 2 | 86 | Strong |
| Filaire et al. [85] | 2 | 2 | 2 | 2 | 2 | 2 | 1 | 2 | 1 | 2 | 2 | 91 | Strong |
| Filaire & Lac [84] | 1 | 1 | 2 | 2 | 1 | 2 | 1 | 2 | 1 | 2 | 2 | 77 | Strong |
| Foretić et al. [109] | 2 | 2 | 2 | 2 | 2 | 2 | 2 | 2 | 1 | 2 | 2 | 95 | Strong |
| Foretić et al. [115] | 2 | 2 | 2 | 2 | 2 | 2 | 2 | 2 | 1 | 2 | 2 | 95 | Strong |
| García-Ceberino et al. [111] | 2 | 2 | 2 | 2 | 2 | 2 | 2 | 2 | 1 | 2 | 2 | 95 | Strong |
| Gonzalez-Bono et al. [89] | 2 | 2 | 2 | 2 | 2 | 2 | 1 | 2 | 1 | 2 | 1 | 86 | Strong |
| Izquierdo & Redondo [79] | 1 | 2 | 2 | 2 | 2 | 2 | 2 | 2 | 2 | 2 | 2 | 95 | Strong |
| Izquierdo & Redondo [107] | 2 | 2 | 2 | 2 | 2 | 2 | 2 | 2 | 1 | 2 | 2 | 95 | Strong |
| Juliff et al. [75] | 2 | 2 | 2 | 2 | 2 | 1 | 2 | 2 | 1 | 2 | 2 | 91 | Strong |
| Kamińska et al. [100] | 2 | 1 | 1 | 2 | 2 | 2 | 2 | 2 | 1 | 2 | 1 | 82 | Strong |
| Karaca et al. [72] | 1 | 1 | 2 | 2 | 2 | 2 | 2 | 2 | 1 | 2 | 2 | 86 | Strong |
| Kocabaş et al. [70] | 2 | 2 | 2 | 2 | 2 | 2 | 2 | 2 | 1 | 2 | 2 | 95 | Strong |
| Kostopoulos et al. [106] | 2 | 2 | 2 | 2 | 2 | 2 | 2 | 2 | 1 | 2 | 1 | 91 | Strong |
| Koyama et al. [102] | 2 | 2 | 2 | 2 | 2 | 2 | 2 | 2 | 1 | 2 | 2 | 95 | Strong |
| Künstlinger et al. [60] | 0 | 1 | 2 | 1 | 1 | 1 | 0 | 1 | 0 | 1 | 1 | 41 | Weak |
| Leite et al. [125] | 2 | 2 | 2 | 2 | 2 | 2 | 1 | 2 | 2 | 2 | 2 | 95 | Strong |
| Lima et al. [140] | 2 | 1 | 2 | 2 | 2 | 1 | 2 | 2 | 1 | 2 | 2 | 86 | Strong |
| Liveris et al. [81] | 2 | 1 | 2 | 2 | 2 | 2 | 2 | 2 | 2 | 1 | 2 | 91 | Strong |
| Lukonaitienė et al. [147] | 2 | 2 | 2 | 2 | 2 | 2 | 2 | 2 | 1 | 2 | 2 | 95 | Strong |
| Marcelino et al. [124] | 2 | 2 | 2 | 2 | 2 | 1 | 2 | 2 | 1 | 2 | 2 | 91 | Strong |
| Marin et al. [71] | 2 | 2 | 2 | 2 | 2 | 2 | 2 | 2 | 1 | 2 | 2 | 95 | Strong |
| Mariscal et al. [73] | 2 | 2 | 2 | 2 | 2 | 2 | 2 | 1 | 1 | 2 | 1 | 86 | Strong |
| Martinez-Amat et al. [93] | 2 | 2 | 2 | 2 | 2 | 2 | 2 | 2 | 1 | 2 | 2 | 95 | Strong |
| Milioni et al. [80] | 2 | 2 | 2 | 1 | 2 | 1 | 2 | 2 | 1 | 2 | 2 | 86 | Strong |
| Moreira et al. [67] | 2 | 2 | 2 | 2 | 2 | 1 | 2 | 2 | 1 | 2 | 2 | 91 | Strong |
| Moreira et al. [94] | 1 | 2 | 2 | 2 | 2 | 1 | 1 | 2 | 2 | 2 | 2 | 86 | Strong |
| Moreira et al. [64] | 2 | 2 | 2 | 2 | 2 | 1 | 1 | 2 | 2 | 2 | 2 | 91 | Strong |
| Moreira et al. [95] | 2 | 2 | 2 | 2 | 2 | 2 | 2 | 2 | 1 | 2 | 2 | 95 | Strong |
| Moreira et al. [90] | 2 | 2 | 2 | 2 | 2 | 1 | 2 | 2 | 1 | 2 | 2 | 91 | Strong |
| Moreno-Pérez et al. [51] | 2 | 2 | 2 | 2 | 2 | 2 | 1 | 1 | 1 | 1 | 0 | 73 | Moderate |
| Moreno-Villanueva et al. [137] | 2 | 2 | 2 | 2 | 2 | 2 | 2 | 2 | 1 | 2 | 2 | 95 | Strong |
| Mortatti et al. [144] | 2 | 2 | 2 | 2 | 2 | 2 | 2 | 2 | 1 | 2 | 2 | 95 | Strong |
| Nemet et al. [135] | 2 | 2 | 2 | 2 | 2 | 2 | 2 | 2 | 1 | 2 | 2 | 95 | Strong |
| O’Donnell et al. [157] | 2 | 1 | 2 | 2 | 2 | 1 | 2 | 2 | 2 | 2 | 2 | 91 | Strong |
| Obmiński et al. [52] | 2 | 1 | 0 | 1 | 1 | 0 | 0 | 0 | 1 | 1 | 1 | 36 | Weak |
| Obmiński et al. [61] | 1 | 1 | 2 | 1 | 2 | 1 | 1 | 2 | 1 | 1 | 2 | 68 | Moderate |
| Parthimos et al. [53] | 1 | 1 | 1 | 2 | 2 | 1 | 1 | 2 | 1 | 2 | 2 | 73 | Moderate |
| Peñailillo et al. [145] | 2 | 2 | 2 | 2 | 1 | 2 | 2 | 2 | 1 | 2 | 2 | 91 | Strong |
| Perrea et al. [54] | 1 | 1 | 2 | 2 | 1 | 2 | 2 | 2 | 1 | 1 | 1 | 73 | Moderate |
| Pinto et al. [139] | 2 | 1 | 2 | 2 | 1 | 1 | 2 | 2 | 1 | 2 | 2 | 82 | Strong |
| Pinto et al. [142] | 2 | 2 | 2 | 1 | 2 | 1 | 2 | 2 | 1 | 2 | 2 | 86 | Strong |
| Pliauga et al. [77] | 2 | 2 | 2 | 2 | 2 | 1 | 2 | 2 | 1 | 2 | 2 | 91 | Strong |
| Ravier et al. [121] | 2 | 2 | 2 | 2 | 2 | 2 | 2 | 2 | 1 | 2 | 2 | 95 | Strong |
| Ribeiro et al. [138] | 2 | 2 | 2 | 2 | 2 | 2 | 2 | 1 | 1 | 2 | 2 | 91 | Strong |
| Ronglan et al. [156] | 2 | 2 | 2 | 2 | 2 | 1 | 1 | 2 | 1 | 2 | 2 | 86 | Strong |
| Ruggiero et al. [151] | 1 | 1 | 2 | 2 | 2 | 1 | 2 | 2 | 1 | 2 | 2 | 82 | Strong |
| Russell et al. [69] | 2 | 2 | 2 | 2 | 2 | 2 | 2 | 2 | 1 | 2 | 2 | 95 | Strong |
| Russell et al. [146] | 2 | 2 | 2 | 2 | 2 | 1 | 2 | 2 | 1 | 2 | 2 | 91 | Strong |
| Sánchez et al. [150] | 2 | 2 | 2 | 2 | 2 | 2 | 2 | 2 | 1 | 2 | 2 | 95 | Strong |
| Sansone et al. [130] | 2 | 2 | 2 | 2 | 2 | 1 | 2 | 2 | 1 | 2 | 2 | 91 | Strong |
| Sansone et al. [152] | 2 | 2 | 2 | 2 | 2 | 2 | 1 | 1 | 1 | 2 | 2 | 86 | Strong |
| Sepahvand et al. [55] | 1 | 2 | 2 | 1 | 1 | 1 | 1 | 1 | 1 | 1 | 2 | 64 | Moderate |
| Setuain et al. [56] | 2 | 1 | 2 | 2 | 2 | 1 | 2 | 0 | 1 | 1 | 2 | 73 | Moderate |
| Song [62] | 1 | 0 | 0 | 0 | 0 | 2 | 0 | 1 | 0 | 0 | 0 | 18 | Weak |
| Souglis et al. [103] | 2 | 2 | 2 | 2 | 2 | 2 | 1 | 2 | 2 | 1 | 2 | 91 | Strong |
| Sybil et al. [63] | 1 | 0 | 1 | 1 | 1 | 1 | 0 | 1 | 0 | 1 | 1 | 36 | Weak |
| Tharp [57] | 1 | 2 | 2 | 1 | 2 | 2 | 1 | 2 | 1 | 1 | 1 | 73 | Moderate |
| Tsarbou et al. [101] | 1 | 2 | 2 | 2 | 2 | 2 | 1 | 1 | 1 | 2 | 1 | 77 | Strong |
| Turgut et al. [58] | 2 | 1 | 1 | 2 | 2 | 2 | 1 | 2 | 0 | 1 | 2 | 73 | Moderate |
| Vitale et al. [92] | 2 | 2 | 2 | 2 | 2 | 2 | 2 | 2 | 2 | 2 | 2 | 100 | Strong |
| Wang et al. [59] | 2 | 1 | 2 | 1 | 2 | 2 | 0 | 2 | 1 | 2 | 1 | 73 | Moderate |
| Wedin & Henriksson [99] | 2 | 2 | 2 | 2 | 2 | 2 | 1 | 2 | 1 | 2 | 2 | 91 | Strong |
| Wilke et al. [127] | 2 | 2 | 2 | 2 | 2 | 2 | 2 | 2 | 1 | 2 | 2 | 95 | Strong |
| Wilke et al. [131] | 2 | 2 | 2 | 2 | 2 | 1 | 2 | 2 | 1 | 2 | 2 | 91 | Strong |
| A score of 2 indicates ‘yes’, 1 indicates ‘partial’, and 0 indicates ‘no’. Quality scores: ≥ 75% strong quality, 56–74% moderate quality, ≤ 55% weak quality. | | | | | | | | | | | | | |

| **S2: Supplementary Table 2. Characteristics, intervention details, fatigue categories assessed and key findings of 'match’ studies (n = 61)** | | | | | | | | |
| --- | --- | --- | --- | --- | --- | --- | --- | --- |
| Basketball only match studies (n = 26) | | | | | | |  | |
| Study | Sex/Cohort/sample size (n) | Participant Characteristics: age, height, body mass | Sporting participation details | Measurement schedule | Fatigue Categories assessed | Key findings | |  |
| Abdelkrim et al. [83] | Male, elite u19, n = 38 | 18.2 ± 0.5 yrs 189 ± 5 cm 80.3 ± 6.7 kg | Six basketball matches played by 6 teams (a single match each). 4 x 10 min quarters with a 15 min halftime and 2 min in-between quarters. | Pre-match, At half time, Immediately post-match | Endocrine | - Cortisol was significantly increased at both half-time and post-match (P < 0.05). | |  |
| Akhundov et al. [97] | Male, elite u20, n = 10 | 17.2 ± 1.1 yrs 90.5 ± 12.3 kg 198.4 ± 9.4 cm | Four basketball matches. Each participant was analysed for only a single basketball match. | Pre-match, Immediately post-match | Physical | - Decreased peak force of the biceps femoris long head (P = 0.006) and semimembranosus (P = 0.032) post-match. - Timing of peak force, and peak fibre velocity were not altered in any of the hamstring and adductor muscles. | |  |
| Caprino et al. [87] | Male, club adolescent (u17), n = 10 | 16 ± 1 yrs 183.6 ± 7.0 cm 76.6 ± 8.0 kg | Three basketball matches. Each participant was assessed in only one match with a maximum of four participants per match. | Pre-match, At half time, Immediately post-match | Physical | - Post-match values for total repeated sprint test time and ideal time were significantly worse than baseline (n^2^ = 0.57) and half-time values (n^2^ = 0.58). - Performance decrement was unaffected by the match. | |  |
| Castagna et al. [119] | Male, club adolescent, n = 20 | 16.8 ± 2 yrs 181.7 ± 6.9 cm 72.4 ± 11.4 kg | A single basketball match. 2x10 min halves. | Pre-match, Immediately post-match | Physical | - Jump height and short sprint ability were unaffected by match participation. - Repeated sprint ability was significantly reduced post-match (P < 0.05). | |  |
| Chatzinikolaou et al. [118] | Not stated, elite, n = 10 | 23.2 ± 2.5 yrs 196 ± 5.1 cm 92.9 ± 7.8 kg | A single basketball match followed by a 6-day standard weekly microcycle with training each day. Only measures taken up to 48 H post were extracted for this review. | Baseline conducted during the two days pre-match, 2 H post-match, 24 H post-match, 48 H post-match | Physical, oxidative stress, muscle damage, endocrine, immunology and inflammation, perceptual | - Increased inflammatory markers (ES range = 3.1 - 5.6) and perceived muscle soreness (ES = 8.8) post-match - Decrease in physical performance (ES = 0.5) post-match. - Majority of disturbances peaked within 24 - 48 H post-match before returning to baseline levels. | |  |
| Chavez et al. [50] | Male, recreation, n = 1 | 24 yrs BMI= 25.1 No height or body mass given | Two 'pick-up style' basketball games. 5 vs 5 players per team. First to 30 points wins with 5 min rest between games. Total playing time of 45 min and 6 sec. | Pre-match, Immediately post-match | Physical | - All landing kinematic variables were significantly altered (P < 0.05) post-match. | |  |
| Conte et al. [113] | Male, sub-elite, n = 7 | 20.8 ± 1.6 yrs 195 ± 5.4 cm 88.3 ± 4.2 kg | Six basketball matches across 3 weeks. Two matches per weekend. One on Friday (18:00) and Saturday (16:00). | In the morning before each game | Perceptual | - Perceived fatigue increased the morning post-match (P < 0.001; ES = 1.31) while - Total well-being decreased the morning post-match (P < 0.001; ES = 0.59) - Muscle soreness, mood and stress were unaffected. - Changes did not affect the workload in subsequent game performance. | |  |
| Cortis et al. [91] | Male, club adolescent (u16), n = 10 | 15.7 ± 0.2 yrs No height or body mass given | A Single basketball match. 4 x 10 min quarters with 2 min recovery. | Pre-match, Immediately post-match | Physical | - No changes to grip strength and jumping ability post-match (p > 0.05) - Interlimb co-ordination improved at 180 bpm (P = 0.015). - 10 m sprint performance decreased post-match (P < 0.05, ES = 0.061 - 0.38) | |  |
| de Arruda et al. [98] | Male, elite adolescent (u15, u16 & u17), n = 25 | U15: 14.1 ± 0.3 yrs 186.6 ± 6.9 cm 78.3 ± 12.2 kg  U16: 15.2 ± 0.4 yrs. 191 ± 8.1 cm 88.9 ± 13.8 kg  U17: 16.5 ± 0.5 yrs 191.5 ± 7.2 cm 89.7 ± 18.9 kg | Participants were assessed over three semi-final and three finals basketball matches (one for each age group). Players had to have played for at least 15 min to be included in the study. | Pre-match, Immediately post-match | Endocrine | - Testosterone increased at post-match (P < 0.001). - No difference found between semi-finals and finals matches (P = 0.2). | |  |
| de Arruda et al. [66] | Male, Elite adolescent (u16 & u17), n = 14 | u16:  15.1 ± 0.3 yrs 190.3 ± 9.1 cm 90.4 ± 15.5 kg  u17: 16.5 ± 0.5 yrs 191.5 ± 7.2 cm 89.7 ± 18.9 kg | Four basketball matches (two per age group). One finals match + one regular competition. | Pre-match, Immediately post-match | Endocrine, immunology and inflammation | - Significant increase in testosterone, cortisol and alpha-amylase post-match regardless of match type (P < 0.001). - No change in interleukin-1ß. | |  |
| de Arruda et al. [86] | Male, elite u19, n = 18 | 17.8 ± 0.4 yrs 190 ± 10 cm 87 ± 8.5 kg | Two basketball matches Separated by 8 weeks. One home and one away. | Pre-match, Immediately post-match | Endocrine | - Testosterone and cortisol significantly increased after both home and away matches (P < 0.05). | |  |
| Delextrat et al. [65] | Male, university, n = 9 | 22.8 ± 2.2 yrs 191.3 ± 5.8 cm 88 ± 10.3 kg | A single basketball match. 4 x 10 min quarters. | Pre-match, Immediately post-match | Physical | - Significant reduction in repeated sprint average speed (ES = 0.27, P = 0.03) and horizontal and vertical forces (ES = 0.24 & 1.61; P < 0.05) post-match - No alterations to maximal sprinting speed or acceleration. - Significant increase in contact time, stride duration, and stride frequency (P < 0.05). | |  |
| Díaz-Castro et al. [110] | Not stated, adolescent, n = 14 | 17.1 ± 3.4 yrs 177 ± 5 cm 72.5 ± 13.7 kg | A single basketball match consisting of 4 x 10 min quarters separated by 5 min breaks. | Pre-match, Immediately post-match | Physical, muscle damage | - CK increased post-match (38.1 %) - CMJ performance decreased (10.8 %). - Changes in CK and CMJ performance were not related to each other (r = -0.1) | |  |
| García-Ceberino et al. [111] | Female, club adolescent, n = 12 | 14 ± 1.4 yrs 167 ± 5 cm 57.2 ± 5.9 kg | A single basketball match. Quarter finals of the Spanish Cadet Championship. | Pre-match, Immediately post-match | Heart rate variability, perceptual | - Significant decrease in heart rate variability post-match (RMSSD: P < 0.05, ES = 2.58). - Anxiety measures were not significantly altered post-match. | |  |
| Gonzalez-Bono et al. [89] | Not stated, elite, n = 16 | Winners: 23.6 ± 1.2 yrs 195.8 ± 2 cm 93 ± 3.8 kg  Losers: 22.9 ± 1.8 yrs 195.4 ± 2.6 cm 94.6 ± 3.5 kg | A single basketball match played between 12:15 and 13:40. | Pre-match, Immediately post-match | Endocrine, perceptual | - Cortisol was significantly increased in both winners and losers post-match (P < 0.02). - Changes in testosterone were not significant (p > 0.05). - Anger, depression, confusion, fatigue, and total mood score were negatively affected by matches in both groups (P < 0.03). | |  |
| Izquierdo & Redondo [107] | Female, club adolescent, n = 68 | 16.5 ± 0.9 yrs 177 ± 5.5 cm 67.5 ± 7.3 kg | Twelve basketball matches. Seven participants assessed per match. | Pre-match, at halftime, immediately post-match | Physical | - Negative changes in sprinting and jumping ability at half-time and full-time for both age groups. - ES for under-16s ranged from 0.02 - 1.09. - ES for under-18s ranged from 0.12 - 1.13. | |  |
| Izquierdo & Redondo [79] | Female, regional adolescent, n = 37 | 17.2 ± 0.4 yrs 177.5 ± 5.4 cm 67.1 ± 8.2 kg | Twelve basketball matches. Seven participants assessed per match. | Pre-match, at halftime, immediately post-match | Physical | - 20 m sprint and CMJ performance decreased in all playing positions post-match (ES range = 0.69 - 1.18). - Change of CMJ was greater in forwards vs. guards and centres. | |  |
| Kostopoulos et al. [106] | Not stated, elite, n = 18 | 24 ± 4 yrs  199 + 9 cm 97 kg | A single basketball match. Played between 17:00-19:00. | Morning of the match. Two consecutive mornings after match (15 H, 39 H post-match) | Muscle damage, immunology and inflammation | - CK only increased post-match in half of the players (P = 0.029) - Returned to baseline by 39 H post-match. - Significant increase in white blood cell count and platelets at 15 H post-match (P < 0.01). | |  |
| Koyama et al. [102] | Male, university, n = 21 | 20 ± 1 yrs 184 ± 11 cm 82.0 ± 11.5 kg | Three basketball matches. 4 x 10 min quarters. Average of 24.5 min participation per participant per match. | Pre-match, 24 H post-match | Muscle damage | - CK increased by an average of 56 % post-match (ES = 0.96). - Increase in CK was significantly correlated with the number of high acceleration movements conducted by the players during the matches (r = 0.65 - 0.74) | |  |
| Liveris et al. [81] | Male, University, n = 16 | 20.2 ± 1.2 yrs 182 ± 10 cm 77.8 ± 11 kg | A single basketball match. 4 x 10 min quarters. | Pre-match, immediately post-match | Physical | - Vertical jump decreased (P = 0.002, ES = 0.9) - Number of landing errors increased post-game (P = 0.034, ES = 0.5) | |  |
| Moreira et al. [90] | Female, elite, n = 11 | 27.4 ± 4.8 years 179.5 ± 5.5 cm 72.0 ± 7.8 kg | A single basketball match. | Pre-match, Immediately post-match  24 H post-match, 48 H post-match | Physical, muscle damage, perceptual | - Small increases in markers of muscle damage and perceived soreness post-match (P < 0.05). - No changes in strength or speed in players. | |  |
| Moreira et al. [94] | Male, elite u19, n = 10 | 19 ± 0.6 yrs 193 ± 6 cm 87 ± 7 kg | Five basketball matches (three practice and two official league matches) played during a 15-week season. Each match was 4 x 10 min quarters with 30 min warm up. | Pre-match, Immediately post-match | Endocrine, immunology and inflammation | - Cortisol significantly increased after official league matches (P < 0.05) but not practice matches. - No changes to salivary immunoglobulin-A after both match types. | |  |
| Moreira et al. [64] | Male, professional, n = 10 | 26.4 ± 3.8 yrs 196 ± 10 cm 100 ± 14 kg | Four basketball matches (two practice and two official league matches). Each match was 4 x 10 min quarters with 30 min warm up. | Pre-match, Immediately post-match | Endocrine | - Cortisol significantly increased after official league matches (P < 0.05) and not practice matches. - Significant positive relationship between RPE and cortisol during official matches (r = 0.75, P < 0.01). | |  |
| Moreno-Pérez et al. [51] | Male, semi-professional, n = 30 | 21.8 ± 4.7 yrs 193.9 ± 8.4 cm 90.4 ± 13.7 kg | Six basketball matches played between February and April. Players were from three separate teams. | Pre-match, immediately post-match,  48 H post-match | Physical | - Ankle dorsiflexion ROM and CMJ performance was increased immediately post-match (ES range = 0.46-0.88). - Ankle ROM experienced small decreases 48 H post-match (ES range = 0.22-0.33). - CMJ was not measured at 48 H post | |  |
| Perrea et al. [54] | Male, club adolescent, n = 13 | Age range = 16-17 years BMI = 20.7 + 2.5 No body mass or height given | A single basketball match. | Pre-match, Immediately post-match | Oxidative stress | - Players demonstrated an increase of 29 % in total serum peroxide post-match. - Polymorphonuclear elastase decreased by 2.5 % post-match. | |  |
| Pliauga et al. [77] | Male, college, n = 10 | 21.5 ± 1.7 years 192.5 ± 5.4 cm 83.5 ± 8.9 kg | A single basketball match. 4 x 10 min quarters with a 15 min break at half time and 8 min breaks after the first and the third quarters. | Pre-match, During each quarter break (physical markers only), Immediately post-match (physical markers only), 24 H post-match,  48 H post-match | Physical, muscle damage | - 10 m sprint performance was decreased immediately post-match, 24H post and 48 H post (P <0.05). - CMJ performance was at decreased 24 H and 48 H post (P < 0.05). - Muscle damage markers were increased at 24 H and 48 H post (P < 0.05). | |  |
| Volleyball only match studies (n = 10) | | | | | | | |  |
| Edmonds et al. [116] | Female, college, n = 14 | 20.4 ± 1.5 yrs  182.5 ± 8.5 cm 77.6 ± 7.9 kg | Participants were assessed across thirty volleyball games during a season. | Every morning: match day - 1, match day, match day +1 | Heart rate variability, perceptual | - HRV measures were unaffected by matches. - Fatigue and muscle soreness were higher the day after the match (P < 0.05) than on match day. | |  |
| Edwards & Turan [88] | Female, college, n = 14 | Age range 18-22 years No height or body mass given | Two volleyball matches played on consecutive days. | Pre-warm up,  during warm-up, Immediately post-match | Endocrine | - Cortisol and testosterone increased significantly post-match (P < 0.01). - Estradiol levels did not change post-match. | |  |
| Karaca et al. [72] | Female, club, n = 12 | 17.0 ± 1. yrs 171 ± 6 cm 62.1 ± 6 kg | A single volleyball match of 3 sets. | Pre-match, Immediately post-match | Oxidative stress, muscle damage, immunology and inflammation | - Nitric oxide and muscle damage markers (CK and lactate dehydrogenase) significantly increased post-match (P < 0.05). - Total antioxidant status increased post-match (P < 0.05). | |  |
| Kocabaş et al. [70] | Male, professional 2nd league, n = 13 | Median age = 24.0 yrs (iQr, 21.0- 25.3) Median height = 185cm (183-187) Mean body mass = 79.2 ± 4.9 kg | A single volleyball match of 4 sets. Total time (including warm up and cool down) was approximately 95 min. | Pre-match, Immediately post-match | Oxidative stress, muscle damage | - Serum total oxidant status decreased post-match (P = 0.027) indicating increased oxidative stress. - Muscle damage markers such as CK and myoglobin were increased post-match (P < 0.001). | |  |
| Künstlinger et al. [60] | Male and female, elite, male n = 16, female n = 8 | Women: 24.8 ± 2.3 yrs Men: No descriptive stats given | Female players (n = 8) were tested during two matches taking place in 1983/84. Three matches were examined following season but only 1 player studied in those matches. One match for men. | Pre-match, Immediately post-match | Muscle damage, endocrine | - CK was slightly increased for the entire group post-match (10 %). - CK increased by a mean of 70.4 % after the case study matches. - Cortisol increased by an average of 47.7 ± 19.4 % after the case study matches. - Statistical significance was not reported | |  |
| Moreira et al. [95] | Male, elite u19, n = 12 | 19 ± 0.3 yrs 196 ± 6 cm 85 ± 8 kg | Two volleyball matches, one regular season and one finals match played 3 weeks apart. Both played between 18:30 and 20:30 | Pre-match, Immediately post-match | Endocrine, immunology and inflammation | - Cortisol and salivary immunoglobulin-A increased after both match types. - However, the statistical significance of these changes was not reported. | |  |
| Tsarbou et al. [101] | Female, college, n = 14 | 19.3 ± 1.1 yrs 168 ± 4 cm 67.0 ± 12.3 kg | A single volleyball match. 60 min long. | Pre-match, Immediately post-match | Physical | - CMJ height was significantly reduced post-match (P = 0.026; ES = 0.6). - Landing errors were significantly increased (P = 0.047, ES = 0.7). | |  |
| Vitale et al. [92] | Male and female, elite, male n = 12, female n = 12 | 26.0 ± 3.4 yrs No height or body mass given. Not separated between sexes. | A single volleyball night match. | In the mornings 1 H after waking on match day, match day + 1, and match day + 2 | Perceptual | - Perceived recovery was significantly decreased the morning post-match (P = 0.001) - Returned to baseline levels on the 2nd morning post-match. | |  |
| Handball only match studies (n = 10) | | | | | | | |  |
| Caetano Júnior et al. [108] | Male, adult, n = 14 | 22.4 ± 2.3 yrs 184 ± 6 cm 87.3 ± 10.1 kg | A single handball match. 2 x 30 min halves with 5 min recovery and a 20 min warm up. No substitutions during match. | Pre-match, Immediately post-match, 2 H post-match | Endocrine, immunology and inflammation | - No significant changes to either salivary cortisol or immunoglobin-A levels. | |  |
| Chatzinikolaou et al. [117] | Not stated, elite, n = 24 | 22.8 ± 1.4 years 186 ± 4.2 cm 82.2 ± 5.5 kg | Two handball matches conducted over 2 seasons (i.e. one per season). Single handball match per participant. | Baseline conducted during the two days pre-match (no specifications on actual timings), 2 H post-match, 24 H post-match, 48 H post-match | Physical, oxidative stress, muscle damage, endocrine, immunology and inflammation, perceptual | - Redox and inflammatory markers were increased immediately post-match (P < 0.05). - Returned to baseline by 24 H. - Markers of neuromuscular function and muscle damage were altered at 24 H post (P < 0.05). - All variables apart from DOMS leg flexors and ROM of the dominant leg had returned to baseline by 48 H post-match. | |  |
| Filaire & Lac [84] | Female, international, n = 14 | 24.1 ± 2.6 yrs  167.8 ± 5.3 cm 61.0 ± 7.5 kg | A full simulated match consisting of 2 x 30 min halves. 2 H session total including warm up. | 08:00 the morning of the match, pre-match, immediately post-match, 08:00 the morning after (12 H post-match) | Endocrine | - No significant differences in hormone markers post-match (P > 0.05). - Testosterone and DHEA levels were significantly correlated with one another (r = 0.65 - 0.88). | |  |
| Filaire et al. [82] | Female, international, n = 14 | 18.5 ± 1.2 yrs No other details given | A single practice match. 2 x 30 min halves with a 10 min halftime. 2 H session total including warm up. | Morning of the match at 08:00, pre-match, immediately post-match, 30 min post-match, 90 min post-match, 08:00 the morning after the match (12 H post-match) | Endocrine | - Cortisol was significantly increased post-match compared with immediately pre-match (P < 0.05) - The increase was greatest at 8 am the following morning (12 H post-match). - However, the morning post-match was significantly lower than the morning of match day (P < 0.05). | |  |
| Foretić et al. [109] | Male, elite, n = 10 | 24.1 ± 3.1 yrs  188.2 ± 6.4 cm 94.6 ± 9.6 kg | A single handball match. 2 x 30 min halves with a 10 min halftime. | Pre-match, half time, immediately post-match | Endocrine | - Testosterone and alpha-amylase demonstrated a significant effect for match on hormone levels (P < 0.01). | |  |
| Foretić et al. [115] | Male, professional, n = 10 | 24.1 ± 3.2 yrs 188 ± 6 cm 94.6 ± 9.6 kg | A single handball match. Players split into contact and non-contact groups for analysis. | Pre-match, half-time, immediately post-match | Endocrine | - Salivary cortisol levels were greater in contact positions compared with non-contact positions at half-time and post-match (ES = 0.90 and 1.38). - Alpha-amylase concentrations were higher in non-contact players at all timepoints (ES = 0.40 - 1.17). | |  |
| Kamińska et al. [100] | Female, sub-elite, n = 12 | 21 ± 2 yrs 170 ± 5 cm 63.2 ± 4.0 kg | Two practice handball matches. 2 x 30 min halves with a 10 min halftime. | Pre-match, immediately post-match | Immunology and inflammation | - There were no changes to hematocrit post-match. | |  |
| Marin et al. [71] | Male, elite, n = 14 | 25 ± 4.5 yrs 187.7 ± 6.6 cm 95.3 ± 9.8 kg | A single handball match. 2 x 30 min halves with a 10 min half-time. | Pre-match, immediately post-match, 24 H post-match | Oxidative stress, muscle damage, immunology and inflammation | - Oxidative stress increased post-match, indicated by changes in the antioxidant system (ES range = 0.71 - 3.53). - Muscle damage markers increased both immediately (lactate dehydrogenase: ES = 1.36) and at 24 H (CK: ES = 1.52) post-match. - Immune cytokines were altered post-match (ES range = 0.67 - 2.09). | |  |
| Mariscal et al. [73] | Female, elite, n = 21 | 23.0 ± 5.4 yrs 170.2 ± 4.1 cm 67.1 ± 7.5 kg | A single handball match. 2 x 30 min halves with a 15 min halftime. | Pre-match, Immediately post-match | Endocrine, immunology and inflammation | - Salivary cortisol increased (P < 0.01) while immunoglobulin-A decreased (P < 0.01) post-match. - Playing position and time on court were shown to have an impact on these markers. | |  |
| Martinez-Amat et al. [93] | Male, university, n = 13 | 24.5 yrs No other details given | A single university handball match | Pre-match, immediately post-match | Muscle damage | - Increases in muscle damage markers post-match in handballers. - Muscle α-actin significantly increased (P < 0.001) suggesting it is a sensitive marker to detect muscle damage. | |  |
| Futsal only match studies (n = 10) | | | | | | | |  |
| Barranco et al. [78] | Male, university, n = 11 | 21.6 ± 1.2 yrs 173 ± 4 cm 74.2 ± 5.1 kg | A single futsal match. 2 x 20 min halves. | Morning of the match after overnight fast, 30 min post-match, 12 H post-match, 36 H post-match, | Muscle damage, immunology and inflammation | - Serum markers of muscle damage increased significantly at the majority of time-points post-match (P > 0.05). - No significant correlations between salivary and serum muscle damage markers (P > 0.05). | |  |
| Bekris et al. [114] | Male, elite, n = 21 | 27.2 ± 3.8 yrs 177 ± 5.9 cm 73.9 ± 7.5 kg | A single futsal match. Participants were split into 2 groups based on playing time. Short playing group was < 16 min, long playing group was > 16 min. | Pre-match, immediately post-match | Muscle damage, endocrine | - Longer playing time was significantly related with higher creatine kinase (P = 0.003; n^2^ > 0.34), lactate dehydrogenase (P = 0.014; n2 > 0.30), and cortisol levels (P = 0.001; n^2^ > 0.39). | |  |
| Cury-Boaventura et al. [76] | Male, professional, n = 16 | 26.4 ± 3.2 yrs 70.2 ± 6.9 kg 172.8 ± 5.7 cm | A single futsal match. Controlled for 20 min match participation per participant. | Pre-match, immediately post-match | Immunology and inflammation | - A single futsal match increased lymphocytosis. - Indicated by increased phosphatidylserine externalization (P < 0.05), DNA fragmentation (P < 0.05), and CD95 expression (P < 0.05). - Decrease in the lymphocyte activation markers CD25 and CD28 (P < 0.05) post-match. | |  |
| de Arruda et al. [104] | Male, club u20, n = 21 | 19.3 ± 0.7 yrs 174.2 ± 4.1 cm 71.8 ± 7.9 kg | Two futsal matches, one home and one away. Both games played between 18:00 - 20:00. Matches were performed 7 weeks apart. | Pre-match, immediately post-match | Endocrine | - Testosterone and cortisol significantly increased post-match at both home and away venues (P < 0.05). - The T:C ratio decreased, indicating a greater rise in cortisol than testosterone. | |  |
| de Freitas et al. [74] | Male, elite u20, n = 13 | 19.0 ± 1.0 yrs  173.7 ± 5.6 cm  69.4 ± 4.9 kg | A single practice futsal match. Match between two teams of 9 players each, with 2 x 20 min halves and a 10 min halftime break. | Pre-match, immediately post-match, 24 H post-match | Physical, oxidative stress, muscle damage, immunology and inflammation, perceptual | - Markers of inflammation, muscle soreness and muscle damage increased post-match (P < 0.05) - CMJ height decreased post-match (P < 0.5). - CK was only significantly elevated (p < 0.01) at 24 H post-match. | |  |
| de Moura et al. [96] | Male, professional, n = 29 | Goalkeepers: 23 ± 1 yrs, 178 ± 3 cm, 74 ± 2 kg Defenders: 21 ± 1 yrs, 174 ± 1 cm, 69 ± 2 kg Wingers: 22 ± 1 yrs, 169 ± 3 cm, 68 ± 2 kg  Pivots: 25 ± 2 yrs, 173 ± 2 cm, 71 ± 2 kg | A single futsal match. Controlled for 20 min match participation per participant. | Pre-match, immediately post-match | Muscle damage, immunology and inflammation | - There were no statistical comparisons of pre-to-post changes in the athletes. - Blood markers of inflammation and muscle damage were higher in goalkeepers than other positions post a single futsal match (P < 0.05). | |  |
| de Moura et al. [68] | Not stated, elite, n = 16 | 26.4 ± 3.2 years 172.8 ± 5.7 cm 70.2 ± 6.9 kg | A single futsal match. Controlled for 20 min match participation per participant. | Pre-match, immediately post-match | Oxidative stress, muscle damage, immunology and inflammation | - Muscle damage markers CK and lactate dehydrogenase increased post-match (P < 0.05). - Inflammatory markers CRP and interleukin-6 increased post-match (P < 0.05). - Neutrophil function increased post-match, indicated by increased phagocytic capacity (P < 0.05). | |  |
| Milioni et al. [80] | Not stated, professional, n = 10 | 22.2 ± 2.5 yrs 174 ± 7 cm 72.7 ± 8.5 kg | A total of five futsal matches. Only a single futsal game examined per participant, with two participants tested per match. 4 x 10 min quarters with 5 min between quarters and a 10 min halftime. | Pre-match, halftime (finishing kicks only), immediately post-match | Physical, technical | - Maximal isometric peak force and voluntary activation were decreased post-match in professional futsal players (ES = 1.33). - Finishing kick performance was unaffected by the futsal match. | |  |
| Moreira et al. [67] | Male, professional, n = 10 | 24 ± 3 yrs 174 ± 5 cm  73 ± 9 kg | Two futsal games each 7 days apart with the results averaged over the two matches. 4 x 10 min quarters with 5 min recovery between each quarter. | Pre-match, immediately post-match | Immunology and inflammation | - Both salivary immunoglobulin-A absolute concentration and secretion rate were decreased post-match (P < 0.05) | |  |
| Sepahvand et al. [55] | Female, adult, n = 10 | 20 ± 2 yrs 162.7 ± 5 cm 55.2 ± 3.5 Kg | A single futsal match. | Pre-match, immediately post-match | Endocrine | - Salivary cortisol was higher pre-match compared with post-match (P < 0.05). | |  |
| Netball only match studies (n = 3) | | | | | | | |  |
| Fien et al. [112] | Female, sub-elite, n = 12 | 21.2 ± 7.3 yrs 174 ± 6.8 cm No body mass given | Eight matches conducted over 4 weeks. Two matches per weekend. Either on the same day or consecutive days. | Morning of day before the match (match -1), morning of each match, the two mornings after each match (match + 1 & match + 2) | Perceptual | - A significant effect for game status on fatigue (P = 0.003) and muscle soreness (P < 0.001). - Only muscle soreness showed a difference between match day and match + 1 or match + 2. - Mood, wellness, and stress was unaffected by game status. | |  |
| Juliff et al. [75] | Female, international u20, n = 12 | 19.2 ± 0.9 yrs 184.1 ± 6.9 cm 72.8 ± 5.2 kg | A single night-time netball match. The match was 60 min and participants had to have played between 45 - 60 min to partake in the study. | Upon waking, 30 min post-waking, pre-match, immediately post-match, 1 H pre-bedtime, upon waking the next morning. | Endocrine | - No statistical tests performed on pre-to-post changes to show significance. - Cortisol increased post-match before decreasing towards bedtime in netball athletes. - Adrenaline and noradrenaline were decreased in the hours post-match. | |  |
| Russell et al. [69] | Female, elite development, n = 12 | 21.3 ± 2.9 y 180.3 ± 5.5 cm 76.2 ± 7.4 kg | Twelve netball matches. Each 60 min long, 4 x 15 min quarters. Played over 6 weekends. Minimum of 20 H in-between games. | Pre-match, immediately post-match | Perceptual | - Perceived physical fatigue increased post-match (P < 0.01). | |  |
| Floorball only match studies (n = 1) | | | | | | | |  |
| Wedin & Henriksson [99] | Male, elite adult and adolescent, n = 23 | All medians with range.  19 yrs (16–34 yrs) 182 cm (173–194 cm) 75 kg (61–98 kg) | A single floorball match. | Pre-match, immediately post-match 2 H post-match | Muscle damage, immunology and inflammation | - Hematocrit was significantly reduced post-match (P < 0.01). - Myoglobin increased significantly post-match (P < 0.01). | |  |
| Mixed sport match studies (n = 3) | | | | | | | |  |
| Filaire et al. [85] | Female, international, handball n = 13, volleyball n = 7 | Handball: 24.1 ± 4.8 yrs, 167.8 ± 5.3 cm, 61.0 ± 7.5 kg Volleyball: 24.6 ± 2.6 yrs, 179.8 ± 5.5 cm, 69.2 ± 6.3 kg | A single handball or volleyball match. 90 -120 min in duration. | Pre-match, immediately post-match | Endocrine | - Cortisol increased 46 % and 26 % in handball and volleyball players respectively. - No significant changes in DHEA and androstenedione in both sports. | |  |
| Filaire et al. [105] | Female, international, handball n = 13, volleyball n = 7 | Handball: 24.1 ± 4.8 yrs, 167.8 ± 5.3 cm, 61.0 ± 7.5kg Volleyball: 24.6 ± 2.6 yrs, 179.8 ± 5.5 cm, 69.2 ± 6.3 kg | Three practice matches. Each session totalling 2 H in length. Week 1, week 7, and week 16 of a training program. | Pre-match, immediately post-match | Endocrine | - Significant decrease in cortisol and androgen markers in volleyball players post-match (P < 0.05). - Cortisol increased post-handball match in week 1 but was not altered post-match in the following measurements. - No androgen markers were affected by the handball matches. | |  |
| Souglis et al. [103] | Male, elite, basketball n = 18, volleyball n = 18, handball n = 18 | Basketball: 25.1 ± 2.8 yrs, 200.1 ± 3.7cm, 96.5 ± 3.7 kg  Volleyball: 25.1 ± 3.1 yrs, 195.6 ± 3.7 cm, 91.6 ± 3.9 kg Handball: 24.4 ± 3.5 yrs, 187.2 ± 4.8 cm, 85.6 ± 5.2 kg | Single game for each. Basketball match was 100 min (including breaks). Handball match lasted approximately 60 min. Volleyball match lasted approximately 80 min. | Morning of the match, immediately post-match, morning after the match (match + 1 ~ 13 H post-match), 2nd morning after the match (match + 2 ~ 37 H post-match) | Muscle damage, endocrine, immunology and inflammation | - Inflammatory markers peaked immediately post-match in all sports (120 % in basketball and volleyball, 90 % in handball) before returning to baseline by the next morning. - Markers of muscle damage peaked the 13 H post-match and had not recovered by 37 H post (2 - 2.5 fold increase from baseline). - Cortisol peaked immediately post-match (70 %) before returning to baseline by morning. | |  |
| CK: Creatine kinase, CMJ: countermovement jump, DHEA: Dehydroepiandrosterone, DOMS: Delayed onset muscle soreness, HRV: Heart rate variability, RMSSD: Root mean square of the successive differences of R-R intervals, ROM: Range of motion, RPE: rate of perceived exertion. | | | | | | | |  |

| **S3: Supplementary Table 3. Characteristics, intervention details, fatigue categories assessed and key findings of 'training’ studies (n = 23)** | | | | | | | | |  |
| --- | --- | --- | --- | --- | --- | --- | --- | --- | --- |
| Basketball only training studies (n = 7) | | | | | | |  | |  |
| Study | Sex/Cohort/sample size (n) | Participant Characteristics: age, height, body mass | Sporting participation details | Measurement schedule | Fatigue Categories assessed | Key findings | |  |  |
| Doma et al. [128] | Female, elite adult and adolescent, n = 10 | age range = 17–32 yrs 179 ± 7cm  76.7 ± 8.3 kg | A 90 min long training session. Consisted of a dynamic warm-up, shooting practice, structured basketball skills training and full court scrimmage. | Pre-training, 24 H post-training | Physical, muscle damage, perceptual | - CMJ power and repeated sprint ability were negatively impacted (ES = 0.43 & 0.84 respectively). - Markers of muscle damage, perceived DOMS (ES = 1.65) and CK (ES = 1.04), increased. - Jump height and change of direction ability were unaffected (P > 0.05). | |  |  |
| Leite et al. [125] | Male, youth regional, n = 10 | 13.3 ± 0.7 yrs 160.9 ± 9.1 cm 49.0 ± 3.8 kg | Thirteen sessions of SSGs (3v3 and 5v5 formats). Each game was 4 x 4 min with 2 min recovery. Court dimensions made so 21 m squared per player regardless of format. | Pre-training, immediately post-training | Physical | - Both squat jump and countermovement height increased after both the 3v3 and regular 5v5 formats (P < 0.05, ES range = 0.33 - 0.89). | |  |  |
| Marcelino et al. [124] | Male, club u19, n = 12 | 18.6 ± 0.5 yrs 192.6 ± 6.5 cm 88.8 ± 14.5 kg | Two SSG protocols with different court areas (28 x 15m vs. 28 x 9m). 3 vs 3 players. 4 x 4 min quarters. 3 min of active recovery (jogging). | Pre-training, immediately post-training | Physical | - Larger playing area had a small negative effect on best (ES = 0.33) and mean (ES = 0.4) times during repeat sprint performance. | |  |  |
| Parthimos et al. [53] | Male, adolescent, n = 10 | 17.5 ± 0.6 yrs 195 ± 5 cm 74.0 ± 1.5 kg | A single basketball training session. Consisted of 10 min warm-up, 30 min technical-tactical drills, 40 min match play or scrimmage drills and 10 min cool down. | Pre-training, immediately post-training | Oxidative stress | - Total anti-oxidant status was significantly lower post-training (P < 0.01). - Acetylcholinesterase activity and NA+, K+-ATPase activity was greatly increased post-training (P < 0.05). | |  |  |
| Sansone et al. [130] | Male, semi-professional, n = 12 | 21 ± 2 yrs 193.9 ± 7.0 cm 84.8 ± 6.6 kg | Participants participated in four different training sessions consisting of four different types of SSGs. SSGs differed by tactical task (offence/defence) or training regime. | Pre-training, immediately post-training | Endocrine | - Cortisol significantly increased post-training (P < 0.05). - Testosterone increased post long-defensive SSG (p = 0.037; r = 0.47) which had the lowest Player Load - Testosterone decreased post short-offensive SSGs (p = 0.028; r = 0.49) which had the highest Player Load. | |  |  |
| Song [62] | Male, junior, n = 11 | No age, height, weight given. | A single training session described as 'heavy-load training'. | 8 am pre-training, immediately post-training, 24 H post-training | Muscle damage, endocrine | - CK was significantly elevated at only 24 H post-training (P < 0.05). - No significant changes to testosterone and lactate dehydrogenase. - Urea nitrogen increased significantly post-training (P < 0.05). | |  |  |
| Wang et al. [59] | Male, elite, n = 10 | 20.6 ± 1.4 yrs 192.6 ± 0.1 cm 81.9 ± 11.7 kg | A single 2 H long training session. Technical and tactical drills, SSGs, scrimmage (1v1 or 2v2), and conditioning. | Pre-training, immediately post-training | Muscle damage | - Blood muscle damage markers were significantly increased post-training (P < 0.05). | |  |  |
| Volleyball only training studies (n = 6) | | | | | | | |  |  |
| Biondi et al. [123] | Male, international, n = 9 | Age range = 20-30 yrs 184 ± 0.07 cm 68 ± 2.9 kg | A single 2 H training session. | Pre-training 2 H post-training 12 H post-training | Immunology and inflammation | - Leukocyte and polymorphonuclear elastase significantly increased 2 H post-training (P < 0.0001) then returned to baseline levels at 12 H. | |  |  |
| Eliakim et al. [133] | Male and female, adolescent international, female: n = 13, male: n = 14 | Female: 16.0 ± 0.4 yrs, 175.6 ± 1.8 cm, 64.1 ± 1.8 kg  Male: 16.3 ± 0.3 yrs, 190.9 ± 1.2 cm, 77.4 ± 1.4 kg | A single 1 H training session | Pre-training, immediately post-training | Endocrine, immunology and inflammation | - Significant changes in growth hormone and testosterone and proinflammatory cytokine interleukin-6 (P < 0.05). - No significant changes in cortisol and other endocrine hormones. | |  |  |
| Eliakim et al. [136] | Female, elite, n = 13 | 16.0 ± 1.4 yrs 175.6 ± 6.3 cm 64.1 ± 6.5 kg | Two typical volleyball training sessions. 60 min each (warm up, volleyball drills and conditioning). Separated by a seven-week training period. | Pre-training, immediately post-training | Endocrine, immunology and inflammation | - Growth hormone and interleuken-6 significantly increased after both training sessions (P < 0.05). - The interleukin-6 response was significantly reduced after the training period. | |  |  |
| Nemet et al. [135] | Male, elite, n = 14 | 16.3 ± 1.1 yrs 190.9 ± 4.1 cm 77.4 ± 9.7 kg | Two typical volleyball training sessions. 60 min each (warm up, volleyball drills and conditioning). Separated by seven weeks. | Pre-training, immediately post-training | Endocrine, immunology and inflammation | - Growth hormone, testosterone and interleukin-6 were significantly increased post-training (p < 0.05). | |  |  |
| Setuain et al. [56] | Male, elite, n = 12 | 23.7 ± 4.9 yrs  198.1 ± 6.2 cm 92.2 ± 10.3 kg | A single training session. Consisting of SSGs and technical ball skills. Jump volume was controlled for (up to 110 jumps). | Pre-training, immediately post-training | Physical | - Training induced a 10 % decrease in peak vertical ground reaction forces at landing during unilateral CMJs. - Jump height was unaffected. - No bilateral CMJ variables were altered. | |  |  |
| Turgut et al. [58] | Male and female, primary school, female: n = 13, male: n = 14 | Boys: 10.4 ± 0.1 yrs Girls: 10.7 ± 0.9 yrs   Group averages: 39.4 ± 10.3 kg  147.9 ± 8.2 cm | A single 2 H training session. Split into 2 x 1 H with 15 min rest in-between. | Pre-training, immediately post-training | Muscle damage, endocrine | - Urinary proteins, creatinine and insulin-like growth factor-1 increased post-training in both boys and girls (p < 0.05). - The response did not differ between them. | |  |  |
| Futsal only training studies (n = 3) | | | | | | | |  |  |
| Moreno-Villanueva et al. [137] | Male, professional, n = 17 | 23.1 ± 4.5 yrs 175 ± 6 cm 75.5 ± 7.5 kg | Sixteen training sessions conducted during preseason. Average length of 98 ± 7 min | Pre-training, immediately post-training | Physical | - Loaded back squats were significantly reduced post-training (ES range = 1.41 - 1.83). - For all the tests the mean change of the variables was smaller than the minimal detectable change. | |  |  |
| Wilke et al. [127] | Male, elite adult and u21, n = 22 | 21.5 ± 5.2 yrs 174.1 ± 5.6 cm 69.6 ± 7.0 kg | A single 70 min futsal training session. Consisted of high-intensity technical-tactical drills. | Pre-training, immediately post-training, 3 H post-training, 24 H post-training, 48 H post-training, | Physical, muscle damage, perceptual | - Identified different recovery profile clusters: Faster recovery, slower perceptual recovery, and slower physiological recovery. - Faster recovery players exhibited better post-training recovery in 10-m sprint, total quality recovery scale, vigour, and fatigue (P < 0.05). | |  |  |
| Wilke et al. [131] | Male, elite u20, n = 13 | 18.8 ± 1.0 yrs 174 ± 7 cm 67.2 ± 8.5 kg | A single 70 min futsal training session. Consisted of high-intensity technical-tactical drills. | Pre-training, immediately post-training, 3 H post-training, 24 H post-training, 48 H post-training | Physical, muscle damage, perceptual | - CK significantly increased at all timepoints post-training (P < 0.05), peaking at 24 H post-training. - Perceived recovery and mood were negatively impacted post-training but returned to baseline at 24 H. - Physical performance was unchanged. | |  |  |
| Handball only training studies (n = 4) | | | | | | | |  |  |
| Amin et al. [126] | Male, club u21, n = 18 | 19.2 ± 0.3 yrs  BMI = 23. 89 ± 3.26 kg/m2 | A single 90 min training session. Intensity at 60-70 % of match-intensity. Included jogging, passing the ball while standing/jogging and shooting. | Pre-training, immediately post-training | Immunology and inflammation | - Both interleukin-6 and tumour necrosis factor-α decreased (P < 0.001) post-training. White blood cell and platelet count increased (P < 0.01). | |  |  |
| Dello Iacono et al. [129] | Male, elite u21, n = 12 | 19.3 ± 0.4 years 186.5 ± 8.4 cm 86.8 ± 8.4 kg | Two training sessions consisting of 3-a-side SSGs. One non-contact and one contact. 5 x 3 min blocks with 1 min recovery. Balls all around court for immediate availability to prevent stoppages. | Pre-training, immediately post-training | Physical, immunology and inflammation | - Physical contact resulted in greater disturbances to neuromuscular function and inflammation (P < 0.05). - Multiple CMJ and plyometric push-up variables improved post non-contact. - Changes in neuromuscular function correlated with the number of contacts sustained in the contact games (r = 0.971, P < 0.001). | |  |  |
| Djordjevic et al. [122] | Male, adolescent, n = 14 | 19.1 ± 1.1 yrs 183.8 ± 6.6 cm 80.6 ± 9.7 kg | A single 90 min practice session. Technical and tactical drills and scrimmage. | Pre-training, immediately post-training | Oxidative stress | - Serum nitrite and superoxide dismutase were altered post-training (P < 0.05), indicating limited exercise induced oxidative stress. | |  |  |
| Netball only training studies (n = 2) | | | | | | | |  |  |
| Birdsey et al. [120] | Female, elite, n = 14 | 23 ± 4 yrs 180 ± 10 cm 73.2 ± 8.0 kg | A single 90 min netball specific training session. 20 min warm up. Technical and tactical drills + specific match play (3 x 5-8 min). | Pre-training, immediately post-training, 2 H post-training, 24 H post-training | Physical, muscle damage, endocrine, perceptual | - CMJ height (ES = 0.25), testosterone (ES = 1.19) and cortisol (ES = 0.95) were elevated immediately post-training. - At 2 H post-training CK was elevated (ES = 1.87) while mood state was negatively altered (ES = 0.84). - 24 H post training CK (ES = 0.98) was still elevated, and neuromuscular performance was suppressed (ES range = 0.27 - 0.39). | |  |  |
| Birdsey et al. [134] | Female, international adult and u21, n = 11 | 21 ± 1 yrs 181 ± 7 cm 76.8 ± 10.2 kg | Two training days separated by one week. Consisted of a strength and netball session. Conducted in different orders each day (STR-NET vs NET-STR).  Only PRE to POST the netball session on the NET-STR day was extracted. 107 min netball specific session. Technical drills with some contested play. | Pre-training, immediately post-training, 2 H post-training | Physical, endocrine, perceptual | - There were increases in jump performance immediately and 2 H post-training (ES range = 2.4 - 2.8). - Testosterone and cortisol also increased immediately post and 2 H post-training (ES range = 08-1.3). | |  |  |
| Mixed sports training studies (n = 1) | | | | | | | |  |  |
| Bruzda-Zwiech et al. [132] | Male, high school basketball and volleyball, basketball: n = 12, volleyball: n = 17 | Basketball: 16.07 ± 1.7 yrs Volleyball: 17.03±1.01 yrs  No heights or body mass given | A single 90 min standard training session for each sport (volleyball and basketball). | Pre-training, immediately post-training | Endocrine, immunology and inflammation | - Cortisol decreased after both basketball and volleyball practices (P < 0.01). - Salivary alpha-amylase increased post-practice in both groups (P < 0.01). - Salivary immunoglobulin-A increased in the volleyball group (P < 0.05) but not basketball. | |  |  |
| CK: Creatine kinase, CMJ: countermovement jump, DHEA: Dehydroepiandrosterone, DOMS: Delayed onset muscle soreness, HRV: Heart rate variability, NET: Netball, RMSSD: Root mean square of the successive differences of R-R intervals, ROM: Range of motion, RPE: rate of perceived exertion, SSGs: Small-sided games, STR: strength. | | | | | | | |  |  |
| **S4: Supplementary Table 4. Characteristics, intervention details, fatigue categories assessed and key findings of 'tournament’ studies (n = 12)** | | | | | | | | | |
| Basketball only tournament studies (n = 3) | | | | | | | |  | |
| Study | | Sex/Cohort/sample size (n) | Participant Characteristics: age, height, body mass | Sporting participation details | Measurement schedule | Fatigue Categories assessed | Key findings | |  |
| Lukonaitienė et al. [147] | | Female, international u18 & u21, u18: n = 10, u21: n = 11 | u18: 18.0 ± 0.4 yrs 179.9 ± 6.6 cm 70.2 ± 5.1 kg  u21: 20.5 ± 2.9 yrs 178.4 ± 8.8 cm 73.0 ± 9.7 kg | Ten-day tournament. Seven matches in nine days (1st day = rest day). | In the morning of each day upon waking | Heart rate variability, perceptual | - No significant changes in HRV over the course of the tournament (P > 0.05). - Well-being was significantly decreased towards the end of the tournament in the u18s (P < 0.05). - No changes in the u20s. | |  |
| Pinto et al. [139] | | Male, adolescent, n = 10 | 14.8 ± 0.5 yrs 173.7 ± 5.9 cm 59.4 ± 9.1 kg | Three matches over two days. Two matches on day 2. 4 x 8 min quarters. | Pre-match 1, immediately post-match 1 (physical, mood, stress), pre-matches 2 + 3 (recovery + anxiety + mood), post-matches 2 + 3 (Physical + mood + stress) | Physical, perceptual | - Significant changes in the depression, anger, and vigour subscales of mood (P < 0.05, ES range = 1.2 - 1.4) after match 3. - Significant decrease in CMJ (ES = 1.4) and 20 m sprint performance (ES = 1.2) across the competition. - Perceived fatigue and cognitive anxiety remained unchanged. | |  |
| Pinto et al. [142] | | Not stated, university, n = 12 | 20.8 ± 2.1 yrs 184 ± 10 cm 83.2 ± 12.4 kg | Four basketball matches played over three consecutive days. Two matches on the first day. One match each on day 2 and 3. Each match was 4 x 10 min quarters. | All metrics PRE 1st match of the day.  Only questionnaires (but excluding total quality of recover) post the last match of each day. | Physical, perceptual | - CMJ (P = 0.03) and perceived recovery (P = 0.02) were negatively altered over the tournament. - Mood state was impacted by match participation on the day (P < 0.05) but showed no cumulative effects over the course of the tournament. - Stress tolerance was not affected (P > 0.05) | |  |
| Volleyball only tournament studies (n = 3) | | | | | | | | |  |
| Mortatti et al. [144] | | Male, elite, n = 11 | 26.4 ± 4.4 yrs 191.4 ± 8.4 cm 85.1 ± 8.7 kg | Three volleyball matches over three days. Matches were 84, 157, and 99 min in length. | Baseline 4 pm day before the tournament, immediately post each match | Endocrine, perceptual | - Cortisol was higher post each match compared to baseline (P < 0.01). - Cortisol did not differ between matches, suggesting no cumulative effect of the congested fixtures. - Stress tolerance was negatively affected by match participation (x^2^ = 15.06; P < 0.01) and continued to decrease across the matches. | |  |
| Obmiński et al. [52] | | Male, international, n = 10 | None given | A 5-day international tournament. One match each day. All matches played at 17:00 | 08:00 the day before tournament, 08:00 every day of tournament | Muscle damage, endocrine, perceptual | - CK increased progressively over the tournament (P < 0.05). - Testosterone levels remained stable until day 3 before dropping by 23 % (P < 0.05). - Cortisol decreased towards the end of the tournament. - Anxiety was elevated throughout the tournament while perceived well-being was unaffected. | |  |
| Peñailillo et al. [145] | | Male and female, international, female: n = 12, male: n = 12 | Female: 17.0 ± 1.8 yrs, 177 ± 4 cm, 67.9 ± 4.5 kg  Male: 22.0 ± 4.3 yrs, 191 ± 8 cm, 83.5 ± 11.4 kg | Three matches during the group stages of an international tournament. 24 - 48 H in between each match. | 08:00 each morning, pre-match, immediately post each match | Endocrine | - Cortisol increased only after match 2 in men (+53.7 %, P = 0.0003). - Testosterone increased post-match 3 in women (+ 37 %, P = 0.0003) but was unaffected by match play in men. | |  |
| Futsal only tournament studies (n = 3) | | | | | | | | |  |
| Charlot et al. [141] | | Not stated, international, n = 10 | 25.5 ± 3.8 yrs 170 ± 7 cm 70.7 ± 8.6 kg | Four matches played over four consecutive days. 2 x 20 min halves each match. | 15 min before each match each day | Perceptual | - Perceived stress and delayed onset muscle soreness increased over the duration of the tournament (ES range = 0.32 - 0.73). - Sleep quality, fatigue and overall fatigue index were not affected. | |  |
| dos Santos et al. [143] | | Female, adult, n = 14 | 22.9 ± 3.7 yrs 163.1 ± 5.7 cm 58.3 ± 5.7 kg | Four futsal matches played on four consecutive days (24 H in-between each). | Morning of the 1st day, immediately post each match | Muscle damage, endocrine | - No significant increases in cortisol or CK from baseline to post-match 1 and 2. - CK then significantly increased for the remainder of the consecutive matches (> 48 H post-baseline). | |  |
| Ribeiro et al. [138] | | Male, adolescent, n = 10 | 16.9 ± 0.7 yrs 71.0 ± 5.1 kg 174.9 ± 4.3 cm | Three matches in four days each consisting of 2 x 40 min halves and 10 min halftime. 24 H between matches 1 and 2, 48 h between match 2 and 3. | 60 min before each match | Endocrine, heart rate variability, perceived | - No differences in HRV variables, salivary cortisol or anxiety over the 4 days of futsal matches. | |  |
| Handball only tournament studies (n = 1) | | | | | | | | |  |
| Lima et al. [140] | | Male, university, n = 9 | 23 ± 3 yrs 75.3 ± 8.6 kg  176.2 ± 10.5 cm | A five-day handball tournament. Five matches in five consecutive days. | 21:00 the night before competition (baseline), 21:00 at end of each day of competition, no measurement on day 5 | Physical, muscle damage, perceptual | - Significant time effect for all variables over the course of the basketball tournament (P < 0.001). - Larger disturbances in neuromuscular and muscle soreness measures on the 4th day of competition. | |  |
| Netball only tournament studies (n = 2) | | | | | | | | |  |
| Birdsey et al. [148] | | Female, elite, n = 11 | 25 ± 4 yrs 180 ± 10 cm 71.8 ± 7.8 kg | Three matches on three consecutive days. 4 x 15 min quarters. | Morning of each match at approximately 07:30 | Physical, muscle damage, endocrine, perceptual | - Varied recovery profile. - Greater perturbations in perceived well-being (42.8 ± 17.6 % decrease), CK (120.8 ± 33.7 % increase) and testosterone (8.7 ± 11.0 % decrease) following consecutive matches. | |  |
| Russell et al. [146] | | Female, professional, n = 10 | 24.9 ± 4.1 yrs 182 ± 9 cm 77.1 ± 9.4kg | Three-day netball tournament with four matches. | Mornings of the day before the match, match days, and two days after the tournament. | Endocrine, perceptual | - Significant decrease over time for recovery markers of physical performance, emotional recovery, and overall recovery (P < 0.05). - Muscular stress, negative emotional stress and overall stress increased (P < 0.05). - Cortisol decreased over the course of the tournament, before increasing 2 days post-tournament (P = 0.031). - Overall recovery was moderately correlated with COD and sRPE (r = -0.41 and -0.40). | |  |
| COD: Change of direction, CK: Creatine kinase, CMJ: countermovement jump, DHEA: Dehydroepiandrosterone, DOMS: Delayed onset muscle soreness, HRV: Heart rate variability, RMSSD: Root mean square of the successive differences of R-R intervals, ROM: Range of motion, RPE: rate of perceived exertion, SSGs: Small-sided games, sRPE: Session rate of perceived exertion. | | | | | | | | |  |

| **S5: Supplementary Table 5. Characteristics, intervention details, fatigue categories assessed and key findings of 'mixed’ studies (n = 12)** | | | | | | | | |
| --- | --- | --- | --- | --- | --- | --- | --- | --- |
| Basketball only 'mixed' studies (n = 6) | | | | | | |  | |
| Study | Sex/Cohort/sample size (n) | Participant Characteristics: age, height, body mass | Sporting participation details | Measurement schedule | Fatigue Categories assessed | Key findings | |  |
| Arruda et al. [155] | Male, elite u19, n = 12 | 18.6 ± 0.5 yrs 192 ± 7 cm 88.9 ± 14.5 kg | Three matches and one training session. Matches were against an 'easy', 'medium', and 'hard' opponent. 4 x 10 min quarters. Training session was 130 min long, consisting of a 90 min technical and tactical and 40 min scrimmage. | Pre,  immediately post | Endocrine | - Significant increase in cortisol from training and matches under all conditions (P < 0.0001). - Both post-match and pre-match cortisol values were higher in the 'High' match condition than other conditions (P < 0.05). | |  |
| Delextrat et al. [149] | Female, elite, n = 9 | 24.3 ± 4.1 yrs 173.0 ± 7.9 cm  65.1 ± 10.9 kg | One match and four training sessions. Match was 4 x 10 min quarters. Training sessions were 120 min each, mixture of technical skills, tactical drills, and scrimmage. | Pre,  immediately post | Physical | - Significant in lower body strength, CMJ height and sprint performance after training and matches (P < 0.05). - No significant decrease in baseline measures across the weekly microcycle. | |  |
| Sánchez et al. [150] | Female, professional, n = 9 | 22.9 ± 2.4 yrs 172.8 ± 7.7 cm 66.3 ± 10.4 kg | Three training sessions and one match conducted over a training week. Match was 4 x 10 min quarters with 10 min halftime and 2 min between quarters. Training sessions 90 min each; 10 min warm-up, 60 min individual and attacking drills, 20 min game scrimmage. | 09:00 each training/match day, 23:00 each training/match day | Endocrine | - No significant differences were observed between the morning and night-time values on any training or match day. | |  |
| Sansone et al. [152] | Female, sub-elite, n = 13 | 22 ± 3 yrs 171.7 ± 6.3 cm 66.3 ± 7.0 kg | 40 training sessions and 14 matches (two friendly, 12 official league matches) during a 14-week period. | Pre, 12 H post 24 H post | Perceptual | - Perceived recovery was lower at 12 H post training and matches (P < 0.001). No differences at 24 H (P = 0.82) | |  |
| Sybil et al. [63] | Female, adolescent, n = 12 | Age range = 18-19 yrs, no other stats given | Two matches at the beginning and end of a six-month macrocycle.  Two 2 H training session at three timepoints (one week, one month, four and a half months) during the macrocycle. | Pre,  immediately post | Endocrine | - All catecholamines increased by 3 - 4-fold post matches and training. | |  |
| Tharp [57] | Male, primary school and highschool, primary school: n = 27, high school: n = 23 | Youth: Age range = 10-12 yrs Adolescent: Age range = 16-18 yrs | Three training sessions and three matches. | Pre,  immediately post | Immunology and inflammation | - Salivary immunoglobulin-A was significantly increased (P < 0.05) after the majority of matches in both age groups. | |  |
| Volleyball only 'mixed' studies (n = 3) | | | | | | | |  |
| Edwards & Kurlander [154] | Female, university, n = 15 | None given | A single match lasting approximately 80 min. Two training sessions of 1 H and 55 min each. | Pre, mid-warm up (not extracted), immediately post | Endocrine | - Cortisol was significantly increased post-match match and the 2nd training session (P < 0.05). - Testosterone significantly increased post-match and both training sessions (P < 0.05). | |  |
| Obmiński et al. [61] | Male, international, n = 1 | None given | One 2 H training session and one 90 min match. | Pre, immediately post, 30 min post | Muscle damage, endocrine | - Markers of muscle damage (CK and urea) increased post-match, while cortisol decreased. - No statistical tests performed (case study). | |  |
| Ruggiero et al. [151] | Male, university, n = 12 | 20.8 ± 1.6 yrs  197.1 ± 5.6 cm 89.2 ± 7.8 kg | Two matches on consecutive days followed by a single training session on day 3. | Pre each match, immediately post each match, pre training (24 H post matches), on a rest day (48 H post matches, 24 H post training) | Physical | - CMJ height was not significantly altered over the course of the matches and training. - Kinetic variables linked to the breaking phase of the CMJ were altered. | |  |
| Handball only ‘mixed’ studies (n = 1) | | | | | | | |  |
| Ronglan et al. [156] | Female, international, training: n = 7, tournament: n = 8 | Training camp: 23.7 ± 2.1 yrs, 179 ± 4 cm, 72.0 ± 6.3 kg  Tournament 23.1 ± 2.0 yrs, 176 ± 5 cm, 71.2 ± 1.8 kg | A two-part study. Part one: Five-day training camp. Six training sessions in five days. 80 - 120 min per session, technical and tactical drills. Part two: Three-day tournament, one match each day. A training session also conducted in morning before 2nd match. | Part one: Pre, immediately post, no post-test for 5th session  Part two: Pre, immediately post, "Baseline" testing done three days before = greater than 48 H so not extracted | Physical | - All neuromuscular tests decreased over the course of the training camp (2-8 %). - Peak declines taking place on day 2 and 3. - CMJ and 20 m sprint were significantly impaired before the final tournament match in comparison with a baseline taken several days before (P < 0.05). | |  |
| Netball only tournament studies (n = 2) | | | | | | | |  |
| O’Donnell et al. [157] | Female, elite, n = 10 | 23 ± 6 yrs 79.8 ± 8.9 kg | An intensity and duration matched netball match and training session. All testing was conducted within a 7-day period. | Pre, immediately post, at 22:00 (2.5 H post) | Endocrine, perceptual | - Cortisol significantly increased from pre-match to post-match with a large effect (P < 0.001; +354 %, ES > 0.8). - No other statistics were given for pre-post-match comparisons. | |  |
| Mixed sport 'mixed' studies (n = 1) | | | | | | | |  |
| Fernández-Rio et al. [153] | Female, semi-professional and professional basketball, volleyball and handball, basketball: n = 18, volleyball: n = 7, handball: n = 12 | Basketball: 26.4 ± 4.3 yrs Handball: 27.2 ± 5.7 yrs Volleyball: 24.4 ± 2.7 yrs | A single training session and match for each sport. | Pre, immediately post | Perceptual | - There were no significant differences in burnout measures for any sport. | |  |
| COD: Change of direction, CK: Creatine kinase, CMJ: Countermovement jump, DHEA: Dehydroepiandrosterone, DOMS: Delayed onset muscle soreness, HRV: Heart rate variability, RMSSD: Root mean square of the successive differences of R-R intervals, ROM: Range of motion, RPE: rate of perceived exertion, SSGs: Small-sided games, sRPE: Session rate of perceived exertion. | | | | | | | |  |

| **S6: Supplementary Table 6. Physical markers of fatigue utilised by the included studies and the number of studies assessing each marker.** | | |
| --- | --- | --- |
| **Physical quality** | **Fatigue Marker** | **Number of studies** |
| Jumping ability | CMJ | 26 |
|  | Vertical jump | 2 |
|  | Squat Jump (unloaded) | 1 |
|  | Unilateral CMJ | 1 |
|  | Drop jump reactive strength index | 1 |
|  | Plyometric pushup | 1 |
| Landing mechanics | Landing error scoring system (LESS) | 2 |
|  | Jump landing kinematics | 1 |
| Linear speed | 20m Sprint | 5 |
|  | 10m Sprint | 7 |
|  | 10m Sprint bouncing a basketball | 1 |
| Change of direction ability | T-Test change of direction test | 3 |
|  | 505 change of direction test | 1 |
|  | 30m Shuttle Sprint (180◦turn = 15m-15m) | 1 |
|  | 20m Shuttle Sprint (180◦turn = 10m-10m) | 1 |
|  | Basketball change of direction test | 1 |
| Muscular strength | 1 RM Bench Press | 3 |
|  | 1 RM Leg Press | 2 |
|  | Grip strength | 2 |
|  | Isokinetic dynamometer peak torque (of the hamstrings and quadriceps) | 2 |
|  | Loaded back squat velocity | 1 |
|  | Maximal isometric voluntary muscle contractions (Knee extension) | 1 |
| Muscle activation | Electromyography (EMG) | 2 |
|  | Muscle fibre mechanics during change of direction | 1 |
| Repeated sprint ability | Basketball line drill test | 4 |
|  | Repeated sprint test (6x4s) | 1 |
|  | Repeated sprint test (10x30m shuttle run) | 1 |
|  | Repeated sprint test (12x20m) | 1 |
| Mobility and coordination | Knee ROM | 2 |
|  | Ankle Dorsiflexion ROM | 1 |
|  | Hand and Foot Flexion Coordination | 1 |
|  | Modified sit and reach test | 1 |
| Technical | Futsal Finishing Kick Performance | 1 |
| CMJ: Countermovement jump, m: Metre, ROM: Range of Motion, s: Second | | |

| **S7: Supplementary Table 7. Countermovement jump variables utilised by the included studies and the number of studies assessing each variable.** | |
| --- | --- |
| **Countermovement jump variable (n = 33)** | **Number of studies** |
| Jump height - velocity | 9 |
| Jump height - flight time | 16 |
| Jump height (visual analysis with hip marker and camera) | 1 |
| Peak power output | 4 |
| Peak power relative to body mass | 2 |
| Peak velocity | 1 |
| Peak eccentric force | 1 |
| Peak concentric force | 1 |
| Peak force relative to body mass | 1 |
| Peak vertical ground reaction force at landing | 1 |
| Force exerted at the end of the downward movement relative to body mass | 1 |
| Change of force during the braking phase | 1 |
| Preload negative exerted force | 1 |
| Peak concentric power | 1 |
| Propulsive vertical impulse | 1 |
| Mean power of the downward (absolute values) and upward phases | 1 |
| Maximum rate of force development relative to body mass | 1 |
| Maximum rate of power development relative to body mass | 1 |
| Time from jump initiation to peak force | 1 |
| Time from jump initiation to peak power | 1 |
| Duration of the CMJ downward movement | 1 |
| Duration of the CMJ upward movement | 1 |
| Duration of the entire CMJ | 1 |
| Breaking Duration in the downward movement | 1 |
| Lowest velocity of the CMJ downward movement | 1 |
| The minimum of the centre of mass displacement during the CMJ | 1 |
| Displacement of centre of mass during the eccentric phase | 1 |
| Displacement of the centre of mass during the braking phase | 1 |
| Centre of mass displacement value at take-off | 1 |
| Centre of mass displacement during the flight phase | 1 |
| Range centre of mass movement Range during the upward push-off movement | 1 |
| Time integral of the force (minus body weight) exerted | 1 |
| The area under the downward movement portion of the force–velocity trace (negative velocity) | 1 |
| CMJ : Countermovement jump |  |

| **S8: Supplementary Table 8. Physiological markers of fatigue utilised by the included studies and the number of studies assessing each marker.** | |
| --- | --- |
| **Physiological markers of fatigue** | **Number of studies** |
| **Oxidative stress (n = 30)** | |
| Protein carbonyls (PC) | 4 |
| Total antioxidant status (TaS) | 3 |
| Reduced glutathione (GSH) | 3 |
| Oxidized glutathione (GSSG) | 3 |
| Glutathione peroxidase activity (GPX) | 3 |
| Aspartate transaminase (aST) | 2 |
| Alanine transaminase (alT) | 2 |
| Total oxidant status (ToS) | 2 |
| TOS/TAS | 2 |
| Redox status (GSH/GSSG) | 2 |
| Total antioxidant capacity (TAC) | 2 |
| Thiobarbituric acid reactive substances/Index of lipid peroxidation (TBARs) | 2 |
| Superoxide dismutase (SOD) | 2 |
| Nitric oxide | 1 |
| Total serum peroxides (TPS) | 1 |
| Myeloperoxidase (MPO) | 1 |
| Polymorphonuclear elastase (PMN) | 1 |
| Fibrinogen | 1 |
| Catalase activity (CAT) | 1 |
| Malondialdehyde (MDA) | 1 |
| Superoxide anion radical (O2−) | 1 |
| Hydrogen peroxide (H2O2) | 1 |
| Nitrites (NO2 −) | 1 |
| Glutathione reductase activity | 1 |
| Thiols | 1 |
| Erythrocyte membrane acetylcholinesterase (AChE) | 1 |
| Na+,K+-ATPase | 1 |
| Mg2+-ATPase | 1 |
| Total antioxidant activity (FRAP assay) | 1 |
| Advanced oxidation protein products (AOPP) | 1 |

| **Muscle damage (n = 8)** | |
| --- | --- |
| Creatine kinase concentration ([CK]) | 28 |
| Lactate dehydrogenase (LDH) | 10 |
| Urea | 6 |
| Myoglobin (MYO) | 4 |
| Sarcomeric α-actin (ACT) | 1 |
| Urinary protein | 1 |
| Creatinine | 1 |
| Mid-Thigh Circumference | 1 |
| **Endocrine (n = 13)** | |
| Cortisol | 43 |
| Testosterone | 23 |
| Alpha-amylase | 6 |
| T:C ratio | 4 |
| Insulin-like growth factor binding protein-3 | 4 |
| Growth hormone (GH) | 4 |
| Adrenaline | 3 |
| Noradrenaline | 3 |
| Insulin growth factor 1 (Igf-1) | 3 |
| Dehydroepiandrosterone (DHEA) | 3 |
| Androstenedione | 2 |
| Estradiol | 1 |
| Dehydroepiandrosterone sulfate (DHEA-S) | 1 |
| **Immunology and Inflammation Markers (n = 30)** | |
| Interleukin-6 (IL-6) | 12 |
| Hematocrit | 7 |
| Immunoglobulin A (SIgA) | 7 |
| Tumour necrosis factor-alpha (TNF-a) | 6 |
| White blood cell count (WBC) | 6 |
| Interleukin 1-beta (IL-1B) | 5 |
| C-reactive protein (CRP) | 5 |
| Interleukin-1 receptor antagonist (IL-1ra) | 5 |
| Hemoglobin concentration | 4 |
| Red blood cell count (RBC) | 2 |
| Phosphatidylserine externalization of neutrophils | 2 |
| Plasma soluble adhesion molecule 1 (sVCAM-1) | 2 |
| Interleukin-8 (IL-8) | 2 |
| Interleukin-10 (IL-10) | 2 |
| Immunoglobulin A secretion rate | 2 |
| WBC subtype ratios | 1 |
| Percentage of CD4 cells | 1 |
| Percentage of CD8 cells | 1 |
| CD4:CD8 ratio | 1 |
| Neutrophils with DNA integrity | 1 |
| CD95 cell expression | 1 |
| Neutrophil Membrane integrity | 1 |
| CD25 cell expression | 1 |
| CD28 cell expression | 1 |
| CTLA-a4 expression | 1 |
| Platelet count | 1 |
| Plasma soluble Adhesion molecule sP-Selectin F | 1 |
| Phagocytic capacity | 1 |
| Polymorphonuclear elastase (PMN-ELA) | 1 |
| Polymorphonucleated leukocytes (PMN) | 1 |
| **Heart rate variability markers (n = 12)** | |
| Root mean square of the successive differences of R-R intervals (RMSSD) | 5 |
| Log-transformed root mean square of successive R-R intervals (lnRMSSD) | 3 |
| Low frequency component of the heart rate variability spectrum (LF) | 3 |
| High frequency component of the heart rate variability spectrum (HF) | 3 |
| Standard deviation of normal R-R intervals (SDNN) | 2 |
| LF:HF ratio | 2 |
| Time between R intervals (RR interval) | 2 |
| Heart rate variability total power | 1 |
| RR variability from heartbeat to short term Poincaré graph (width) (SD1) | 1 |
| RR variability from heartbeat to long-term Poincaré graph (length) (SD2) | 1 |
| Heart rate variability sample entropy (SampEn) | 1 |
| Percentage of intervals > 50 ms different from the previous interval (pNN50) | 1 |
|  |  |

| **S9: Supplementary Table 9. Perceptual markers of fatigue utilised by the included studies and the number of studies assessing each marker.** | |
| --- | --- |
| **Perceived markers of fatigue (n = 16)** | **Number of studies** |
| Total quality recovery scale (TQR) | 6 |
| Perceived DOMS | 6 |
| Custom wellness questionnaires | 5 |
| Brunel mood scale (BRUMS) | 4 |
| Modified brief assessment of mood (BAM+) | 3 |
| Daily Analysis of Life Demands in Athletes questionnaire (DALDA) | 3 |
| Competitive State Anxiety Inventory–2R (CSAI-2R) | 3 |
| Perceived physical fatigue (visual analogue scale) | 1 |
| Perceived stress (visual analogue scale) | 1 |
| The athlete burnout questionnaire | 1 |
| Hooper index | 1 |
| Standardized Spielberger test for state anxiety | 1 |
| Mental disposition (MD) | 1 |
| Physical disposition (PD) | 1 |
| Profile of mood states (POMS) | 1 |
| Short recovery stress scale (SRSS) | 1 |
| DOMS: Delayed onset muscle soreness | |
